# Uptake of label-free graphene oxide by Caco-2 cells is dependent on the cell differentiation status

**DOI:** 10.1186/s12951-017-0280-7

**Published:** 2017-06-21

**Authors:** Melanie Kucki, Liliane Diener, Nils Bohmer, Cordula Hirsch, Harald F. Krug, Vincenzo Palermo, Peter Wick

**Affiliations:** 1 Laboratory for Particles-Biology Interactions, Empa-Swiss Federal Laboratories for Materials Science and Technology, Lerchenfeldstrasse 5, 9014 St. Gallen, Switzerland; 2 International Research Cooperations Manager, Empa-Swiss Federal Laboratories for Materials Science and Technology, Lerchenfeldstrasse 5, 9014 St. Gallen, Switzerland; 3 Istituto per la Sintesi Organica e la Fotoreattività, Consiglio Nazionale delle Richerche (CNR), Via P. Gobetti 101, 40129 Bologna, Italy

**Keywords:** Graphene oxide, Intestinal epithelium, Enterocytes, In vitro, Caco-2, Cell differentiation, Uptake, Microvilli, Brush border

## Abstract

**Background:**

Understanding the interaction of graphene-related materials (GRM) with human cells is a key to the assessment of their potential risks for human health. There is a knowledge gap regarding the potential uptake of GRM by human intestinal cells after unintended ingestion. Therefore the aim of our study was to investigate the interaction of label-free graphene oxide (GO) with the intestinal cell line Caco-2 in vitro and to shed light on the influence of the cell phenotype given by the differentiation status on cellular uptake behaviour.

**Results:**

Internalisation of two label-free GOs with different lateral size and thickness by undifferentiated and differentiated Caco-2 cells was analysed by scanning electron microscopy and transmission electron microscopy. Semi-quantification of cells associated with GRM was performed by flow cytometry. Undifferentiated Caco-2 cells showed significant amounts of cell-associated GRM, whereas differentiated Caco-2 cells exhibited low adhesion of GO sheets. Transmission electron microscopy analysis revealed internalisation of both applied GO (small and large) by undifferentiated Caco-2 cells. Even large GO sheets with lateral dimensions up to 10 µm, were found internalised by undifferentiated cells, presumably by macropinocytosis. In contrast, no GO uptake could be found for differentiated Caco-2 cells exhibiting an enterocyte-like morphology with apical brush border.

**Conclusions:**

Our results show that the internalisation of GO is highly dependent on the cell differentiation status of human intestinal cells. During differentiation Caco-2 cells undergo intense phenotypic changes which lead to a dramatic decrease in GRM internalisation. The results support the hypothesis that the cell surface topography of differentiated Caco-2 cells given by the brush border leads to low adhesion of GO sheets and sterical hindrance for material uptake. In addition, the mechanical properties of GRM, especially flexibility of the sheets, seem to be an important factor for internalisation of large GO sheets by epithelial cells. Our results highlight the importance of the choice of the in vitro model to enable better in vitro-in vivo translation.

**Electronic supplementary material:**

The online version of this article (doi:10.1186/s12951-017-0280-7) contains supplementary material, which is available to authorized users.

## Background

The actual materials science breakthrough of graphene and graphene-related materials (GRM) led to the establishment of a roadmap for their production and possible applications [1, 2]. Some products based on GRM have already reached commercial production level [3]. Mass production and use of GRM, as well as exposure to humans, will increase over the coming years. Close body applications are most likely to happen [4]. Furthermore, graphene oxide (GO) is considered a potential candidate for biomedical applications such as drug delivery, cancer treatment or neuro-implants [5, 6] and it is to date the best studied GRM with respect to biological systems. For comprehensive risk characterization and potential biomedical application of GRM in-depth understanding of their cellular uptake and possible biological effects is a crucial need [7]. Despite several in vivo studies exploring the distribution of GRM after intravenous application in mice and rats [8], the knowledge about the uptake and fate of these materials at the tissue or cellular level is weak. Some studies provide conflicting results about the cellular uptake of GRM in vitro, summarized in recent reviews [9–11]. The possible mechanisms of uptake are unclear and subject of debate. Next to energy-dependent GRM uptake reported for different cell types [5, 12–14], Li and his colleagues described a razor-blade like passive uptake of graphene flakes [15]. It should be emphasized that GRM can considerably vary in their physicochemical properties, which in turn can extensively influence the GRM-cell surface interaction as well as the amount of cellular uptake, ranging from no uptake to high accumulation of the materials inside the cells. Furthermore, the uptake mechanisms and efficiency can be highly cell-type specific. Yue et al. reported negligible uptake of GO with a lateral size of about 350 nm and 2 µm by four different non-phagocytic cell types next to high intracellular accumulation of both GO samples by murine macrophages [12].

In general, a huge number of studies on inhalative exposure to nanomaterials exist and nano-toxicologists agree that the lungs are the most sensitive entry portals into the human body. The healthy skin, on the other hand, has been demonstrated in many studies to be very tight and to form a highly effective barrier for nanomaterial permeation. For the gastrointestinal tract, however, the lowest number of studies exists and these are very controversial regarding the uptake of nanomaterials into the body tissues [16]. For GO some of the most promising applications could be in composite films for packaging [17] to create new, cheap gas barrier coatings for food or beverages [18]. Therefore it is an important point to obtain clear evidence that GO traces are not harmful upon casual ingestion. Only recently the impact of different GO on undifferentiated Caco-2 cells has been reported [19–21], revealing the formation of reactive oxygen species (ROS) induced by GO as well as a close GO-cell surface interaction. Nevertheless no acute toxicity of the applied GO for an exposure range of 5–80 µg GO/ml and an exposure time up to 48 h could be found [20].

The Caco-2 cell line is the foremost applied cell culture model for the human intestinal mucosa. The cell line is derived from colon adenocarcinoma and applied in different model variants, either as monoculture [22–25] or in co-culture with other cell types of the intestinal barrier and underlying tissue, e.g. goblet cells, endothelial cells or B-lymphocytes [26–29]. Upon reaching confluency Caco-2 cells undergo spontaneous differentiation with extensive morphological and physiological changes leading to tight and polarized enterocyte-like epithelial cell layers with brush border (BB) and tight junction (TJ) formation. The differentiated Caco-2 cell culture model more realistically reflects the mature enterocytes in the human body, from the morphological as well as physiological side. Differentiated Caco-2 cells are applied by the pharmaceutical industry to investigate intestinal drug absorption and transport mechanisms to predict bioavailability of substances for oral drug delivery [23–26]. Nevertheless, undifferentiated Caco-2 cells are also often applied to enable fast and high-throughput screening for potential hazardous substances. Previous cellular uptake studies with Caco-2 cells and other epithelial cell types have predominantly been performed with non-polarized undifferentiated cells. To date it is not clear if 2D materials can be taken up by Caco-2 cells at all and if the differentiation status of the cells has an influence on the cellular uptake behaviour.

Therefore the aim of our study was to obtain a mechanistic understanding of the interaction of GO with different cell surface topographies and cell morphologies of undifferentiated and differentiated Caco-2 cells. To analyse the effects of pure GO we applied label-free GO due to the fact that labelling of GO, especially by attachment of larger ligand and fluorescence dye molecules, can alter the surface properties of the material which in turn can significantly alter the cellular uptake behaviour. To investigate the impact of the lateral size of GO on the cellular uptake, we selected two GO samples with broad but different size distributions, previously applied by Kucki et al. and found a lack of acute toxicity for undifferentiated Caco-2 cells [20]. The GRM we used (Table 1, named with the same codes of Ref. [20] for clarity) are:

**Table 1:** Overview on the physicochemical properties of applied graphene oxides and graphene nanoplatelets

	Graphene oxide		Graphene nanoplatelets
	GO-1	GO-3	GNP
Material source	Commercial	Research	Commercial
Preparation method	Modified hummers method	Modified hummers method	Microemulsion
Starting material	Graphite	Natural graphite	Natural graphite
State as received	Powder	Dispersion	Powder
Lateral dimensions	1–40 µm (SEM)	150 nm ± 44 nm (AFM)	Aggregate size 1–10 µm; mean ~5 µm (SEM)
Number of layers	Single to few layer	Single layer	Aggregate thickness <5 µm
C/O ratio (XPS)	1.7 ± 0.1	1.9 ± 0.1	24.0 ± 2.5
Dispersion in …	Ultra-pure water	Ultra-pure water	0.1 mg/ml sodium cholate in ultra-pure water
Dispersion colour	Brown	Brown	Anthracite
Zeta-potential in ultra-pure water	−39.4 ± 1.3 mV	−43.9 ± 1.4 mV	−62.6 ± 1.9 mV

Overview on the physicochemical properties of the applied label-free graphene-related materials (GRM) as determined by Kucki et al. [20]. Original material acronyms were retained for sake of clarity

1. Commercial graphene oxide nanoplatelets formed by several stacked GO layers and with a large lateral size (up to 40 µm), comparable to cell diameter (GO1).
2. Small GO nanosheets, with lateral size below 1 µm, mostly present in solution as single monatomic sheets (GO3) [30].
3. Commercial, non-oxidized graphene nanoplatelets (GNP), poorly dispersible in water, used as a “worst case” reference.

The different materials tested were chosen to allow a direct comparison and study of the effect of shape and oxidation grade on biological activity. GO1 and GNP have similar shape (rigid nanoplatelets) and mesoscopic lateral size, but different surface chemistry (C/O is 1.7 and 24.0 respectively). GO1 and GO3 have similar surface chemistry but different lateral size and shape: GO1 thicker platelets are more rigid, while GO3 monoatomic sheets can easily deform in solution, or upon action of cellular membrane, as will be described in the following sections. The interactions of Caco-2 cells with these different GRM in dependence of the cell differentiation status was analysed primarily by scanning electron microscopy (SEM) and transmission electron microscopy (TEM). We could clearly demonstrate that undifferentiated Caco-2 cells show considerable cellular uptake of GRM, whereas no GRM uptake could be found for differentiated Caco-2 cells which exhibit an enterocyte-like surface topography and cell morphology.

## Methods

### Graphene-related materials and physico-chemical characterisation

Commercial GO (GO1) and graphene nanoplatelet (GNP) powder were obtained from Cheap Tubes Inc. (112 Mercury Drive, Brattleboro Vermont, 05301, USA). GO3 was produced by modified Hummers method. The materials were applied as received without further pre-treatment as it has been already shown recently that acid-treatment simulating stomach transition did not lead to alterations of the material properties [20]. The detailed physicochemical characterisation and material properties, including Raman and XPS characterization of sheet thickness and oxidation, are described in Kucki et al. [20] and summarized as an overview in Table 1. As experiments were performed with the identical batch and suspension of GO1, GO3 and GNP respectively batch-to-batch variability can be excluded. GO1 powder was pre-dispersed in endotoxin-free (<0.005 EU/ml) ultra-pure water (Millipore-Q) to a concentration of 1 mg GO1/ml. Even if GO1 is supposed to be few-layer graphene oxide from the producer, SEM and TEM measurements (see below) showed that it is mostly composed of thicker platelets, 10 layers or above, indicating that GO1 is a material closer to graphite oxide rather than graphene oxide. Thus, dispersion of the GO1 sheets was improved by short bath-sonication for 10 s (hot spot; Bandelin Sonorex RK156 BH). Conversely GO3, being truly composed of monoatomic sheets was easily used as aqueous dispersion without any visible aggregates and with no need of sonication. GNP, being the less soluble of the materials used due to the low presence of oxidized defects on its surface, was dispersed in water containing 0.1 mg/ml sodium cholate, a natural occurring primary bile acid. All GRM samples were stored under sterile conditions at room temperature and protected from light. GRM stock dispersions were vortexed for at least 10 s before further use.

### Cell culture

Caco-2 cells were obtained from ATCC (ATCC^®^HTB-37™, ATCC, Manassas, VA, USA) and cryogenically-preserved until use. After thawing cells were sub-cultured at least twice prior to experimental use. Cells were cultivated in minimum essential medium (MEM, Sigma-Aldrich, Ref. M2279) supplemented with 10% foetal calf serum (FCS, Sigma-Aldrich, Ref. F9665), 1% l-glutamine (Sigma-Aldrich, Ref. G7513), 1% non-essential amino acids (NEAA, Sigma-Aldrich, Ref. M7145) and 1% penicillin–streptomycin–neomycin (PSN, Sigma-Aldrich, Ref. P4083). Cells were maintained at 37 °C and 5% CO_2_ in humidified atmosphere and routinely sub-cultured twice a week at 70–80% confluence by treatment with 0.5% trypsin–EDTA (Sigma-Aldrich, Ref. T3924).

### Differentiation of Caco-2 cells

Caco-2 cells were seeded on porous membrane supports (12-well) at a seeding density of 250,000 cells/well which corresponds to 2.2 × 10^5^ cells/cm^2^. For SEM analysis and immunofluorescence labelling, cells were seeded on polyethylene terephthalate (PET) membranes (ThinCert™, Greiner bio-one, Ref. 665631, 12-well, 3.0 µm pore size). For TEM analysis cells were seeded on polycarbonate (PC) membranes (Transwell, Corning, Ref. 3402, 12-well, 3.0 µm pore size). Cells were grown for 21 days to obtain mature differentiated monolayer. After 21 days the integrity of the Caco-2 monolayer was controlled by measurement of the transepithelial electrical resistance (TEER) by Epithelial Voltohmmeter (EVOM) with sterilized STX2 electrodes (World Precision Instruments, 175 Sarasota Center Boulevard Sarasota, FL 34240-9258, USA).

### Cell exposure—undifferentiated and differentiated Caco-2 cells

For cell culture experiments GRM stock dispersions of 1 mg GRM/ml were diluted to the respective final GRM concentrations in supplemented cell culture medium (MEM supplemented with 10% FCS, 1% l-glutamine, 1% NEAA and 1% PSN). Caco-2 cells were either differentiated for 21 days as described above or seeded on permeable supports (12-well) at a seeding density of 100,000 cells/well which corresponds to 8.9 × 10^4^ cells/cm^2^ and allowed to adhere for 24 h (referred to as undifferentiated). Thereafter, both undifferentiated and differentiated Caco-2 cells grown on permeable supports were exposed to GRM under identical conditions.

### Scanning electron microscopy (SEM) analysis of Caco-2 cells

Caco-2 cells (undifferentiated or differentiated on porous PET membrane supports) were exposed to GO1, GO3 or GNP dispersed in supplemented cell culture medium at concentrations of 10, 20 or 40 µg GRM/ml (corresponding to 7.4, 14.8 or 29.6 µg GRM/cm^2^) for 24 h. Control cells were exposed to supplemented cell culture medium without GRM. After exposure cells were washed twice in pre-warmed phosphate buffered saline (PBS) and fixed with modified Karnovsky fixation solution [4 g paraformaldehyde (CAS 30525-89-4), 50 ml aqua bidest, 5 ml glutaraldehyde 50% (CAS 111-30-8), 45 ml PBS without glucose and pH 7.4] at room temperature for 1 h. Samples were washed twice in PBS and dehydrated by ascending ethanol series (50–100% ethanol) followed by treatment with hexamethyldisilizane (HMDS, CAS 999-97-3). Samples were dried overnight in a fume hood and stored in a desiccator until transfer to SEM sample holders with conductive adhesive tapes. Samples were sputter-coated (Sputter Coater Leica EM ACE600) with gold–palladium (ratio Au/Pd = 80/20; 10 nm thickness). Analysis was performed with a Hitachi S-4800 SEM operating at 2.0 kV.

### TEM preparation of Caco-2 cells—undifferentiated and differentiated cells

Caco-2 cells (undifferentiated or differentiated on porous PC membrane supports) were exposed to GRM as described above or remained untreated. GO-exposed cells and control cells were fixed in 3% glutaraldehyde in 0.1 M sodium cacodylate buffer and washed in 0.2 M sodium cacodylate buffer. After a post-fixation step in 2% osmium tetroxide in 0.1 M sodium cacodylate buffer samples were dehydrated through a graded ethanol series followed by acetone and finally embedded in Epon resin (Sigma-Aldrich). Ultrathin sections were contrasted with 2% uranyl acetate and lead citrate (Reynolds 1963) before imaged in a Zeiss EM 900 TEM (Carl Zeiss Microscopy GmbH, Germany) at 80 kV.

### Flow cytometry

Undifferentiated Caco-2 cells were seeded in 6-well plates at a seeding density of 100,000 cells/ml (250,000 cells/well or 27,700 cells/cm^2^). After 24 h pre-culture cells at 50–60% confluence were incubated for 24 h with the indicated amount of GRM (10, 20, 40 µg/ml or 2.8, 5.5, 11.1 µg/cm^2^ respectively). Control cells were exposed to supplemented cell culture medium without GRM. Control cells exposed to supplemented cell culture medium containing water or sodium cholate without GRM served as solvent controls. To exclude extracellular GRM from the analysis, medium was removed and the cell-layer was washed two times with pre-warmed PBS. Afterwards cells were harvested at 80–90% confluence by treatment with 0.5% trypsin–EDTA, pelleted by centrifugation (200*g*, 5 min) and re-suspended in PBS for flow cytometry. Changes in side scatter (SS) values were recorded on a logarithmic scale and plotted against linearly recorded forward scatter (FS) values or counts using a Gallios TM flow cytometer and Kaluza software (Beckman Coulter). A total of 10,000 counts were analysed per sample. Side scatter values in flow cytometry indicate the granularity or complexity of a cell and it has been shown previously that e.g. the uptake of titanium dioxide (TiO_2_) nanoparticles (NPs) or ellipsoid-shaped supraparticles results in a dose-dependent shift, i.e. increase, in SS values [31, 32]. According to this method cells associated with GRMs (intra- and extracellular) were gated and quantitatively determined in dependence of their increase in side scatter signal.

## Results

### Physicochemical properties of applied graphene oxide and graphene nanoplatelets

**Fig. 1:**
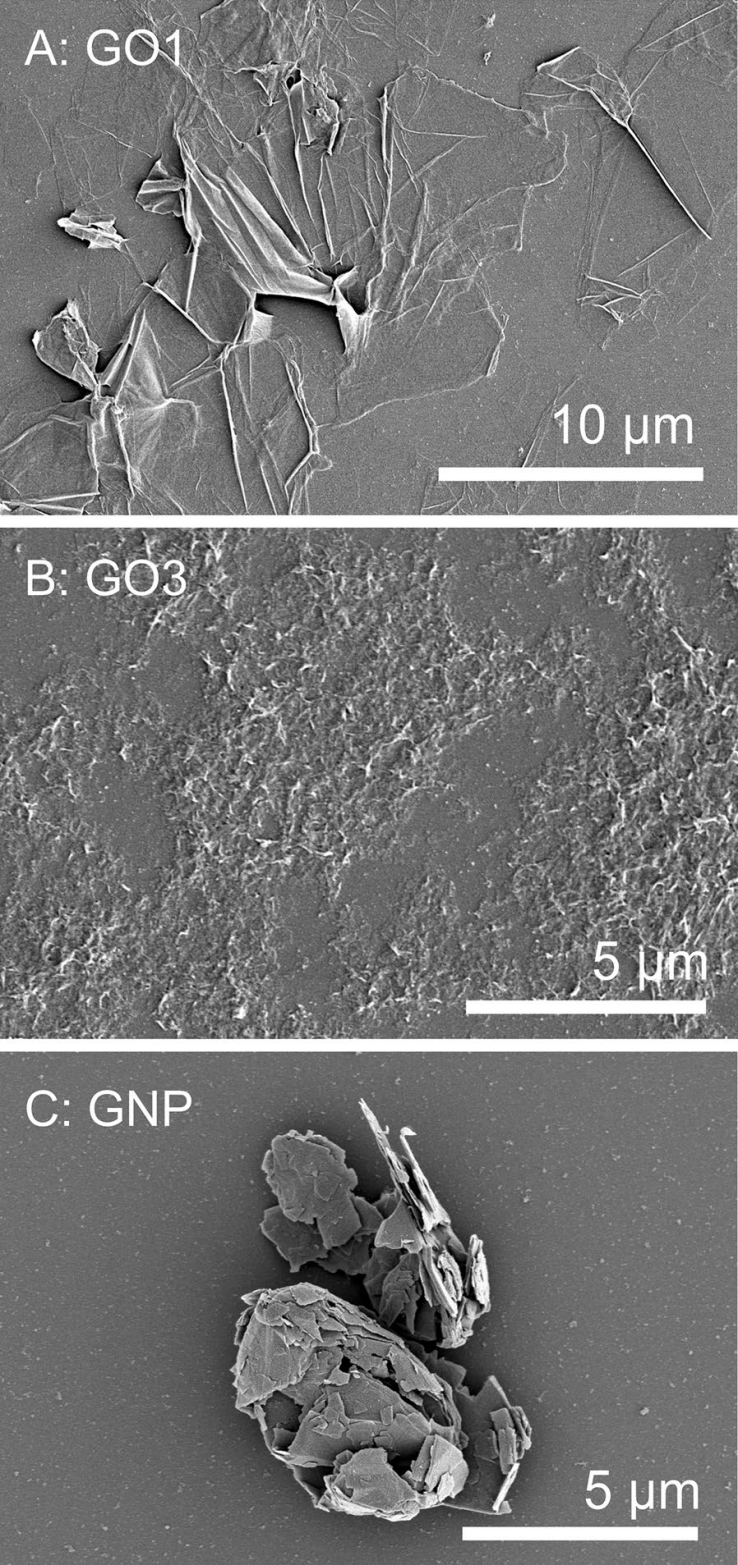
Scanning electron microscopy (SEM) images of applied GO (**A**, **B**) and GNP (**C**). Images show morphology of GRM on glass slides after incubation in supplemented cell culture medium for 24 h in the presence of undifferentiated Caco-2 cells (cells not shown)

As mentioned before, the applied label-free GRM have been already used in a recent study with undifferentiated Caco-2 cells. Details regarding the preparation and characterization of the materials are given in Kucki et al. [20]. Table 1 gives an overview on the physicochemical properties of the applied materials. For sake of clarity and to allow a direct comparison, we retain the material numbering used in Kucki et al. [20]. Both GO samples showed good dispersion behaviour in water due to oxygen-functionalized groups. In a previous study we investigated the potential effect of GO1 and GO3 among other GO on the metabolic activity of undifferentiated Caco-2 cells [20]. This study demonstrated clearly that 24 or 48 h exposure to either GO1 or GO3 in a concentration range from 5 to 80 µg/ml has no acute toxic effect, which is relevant for the investigation of cellular uptake. GNP aggregates, also applied in Kucki et al. [20], were included as ‘reference material’ as further detailed in the “Methods” section. Figure 1 shows representative images of the GRM morphology after incubation in supplemented cell culture medium in the presence of cells after 24 h.

### SEM analysis of the interaction of GO and GNP with the cell surface of undifferentiated Caco-2 cells

**Fig. 2:**
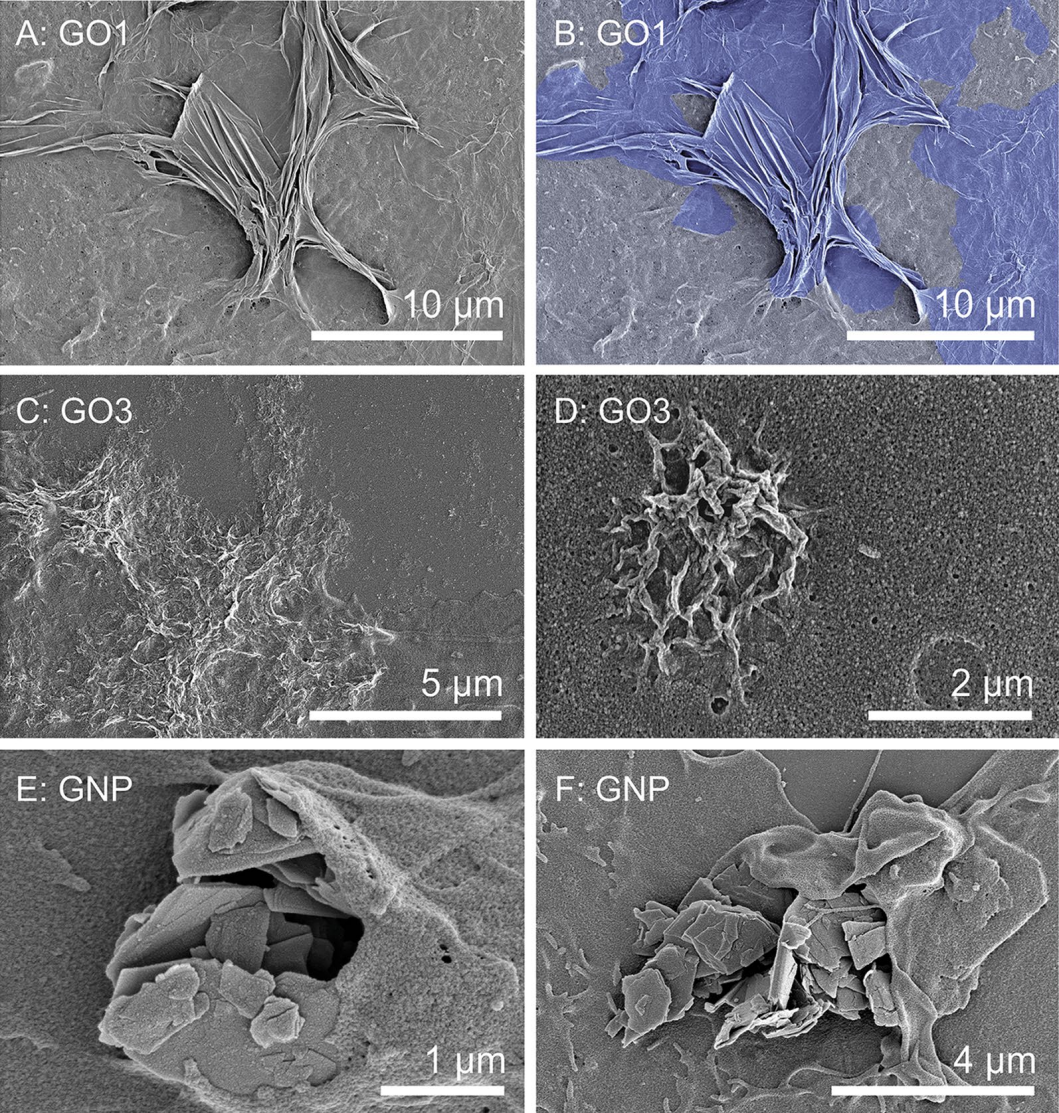
Interaction of GO and GNP with the cell surface of undifferentiated Caco-2 cells. SEM images of undifferentiated Caco-2 cells exposed to either 40 µg/ml GO1 (**A**, **B**), GO3 (**C**, **D**) or GNP (**E**, **F**) for 24 h. **B** Is displayed in false-coloration to enhance visibility of GO1 (*purple*) in contrast to the cell surface (*grey*). GO3 sheets (**C**, **D**) exhibit several folds and show agglomeration forming larger mats of GO on the cell surface. Hints are given towards a simultaneous uptake of several GO3 sheets in form of larger agglomerates. For further SEM images see Additional file 1: Figures S3 and S4

Undifferentiated Caco-2 cells were exposed to different concentrations of either GO1 or GO3 in supplemented cell culture medium. Control cells were cultured in similar medium but were not exposed to GO. Scanning electron microscopy (SEM) analysis of undifferentiated Caco-2 (Fig. 2) cells showed close interaction of the GRM with the cell surface, in agreement with previous results obtained by Kucki et al. [20]. By screening the surface of thousands of Caco-2 cells, we found several hints towards an active uptake of the sheets, or at least towards active attempts of uptake. The active role of the cell membranes was clearly evident in particular for the bulky, rigid GNP, where the cells were strongly deformed trying to engulf the platelets (Fig. 2E, F; Additional file 1: Figure S4). Conversely the thinner, more flexible GO1 platelets were often deformed by mechanical action of the cell membrane, which created multiple folds and bends on the GO1 surface (Fig. 2A, B). At the periphery of Caco-2 islets, cells exhibited wave-like protrusions in contact with GO1 sheets, indicating possible cellular uptake. In contrast, the surface of the centre of these islets showed less amounts of associated GO sheets. As depicted in Fig. 2, cellular protrusions were found at corners and edges of GO1 sheets as well as GNP, covering parts of these GRM. In several cases GO1 exhibited parallel alignment to the cell membrane, similar to observations made by Russier et al. with human and murine macrophages and described as “mask-effect” [33]. The smallest GO3 sheets were instead primarily found in form of mat-like agglomerates of folded and wrinkled sheets as shown in Fig. 2C, D and Additional file 1: Figure S3. The mechanical properties of GO are difficult to estimate as they depend on several factors such as the amount of defects as well as their localization and arrangement in the GO sheets [34]. The pre-requisite for the deformation of the GO sheets by the cell would be that forces generated by the Caco-2 cells are sufficiently high enough to induce folds and wrinkles to the material. Our observations are a clear demonstration of the strong mechanical interactions that can exist between flexible but robust mono- to few layer nano-sheets and human cells.

### TEM analysis of GO uptake by undifferentiated Caco-2 cells

**Fig. 3:**
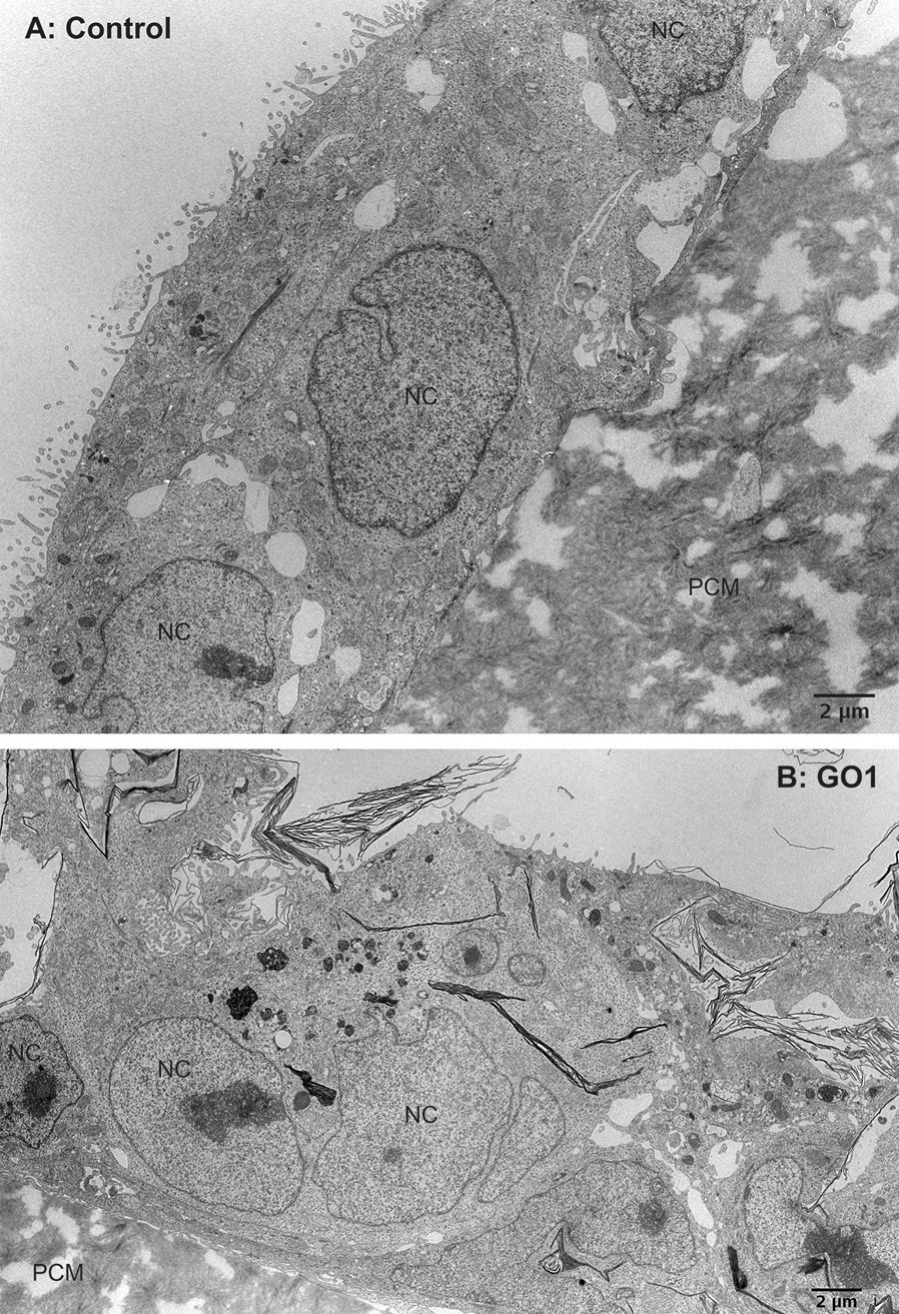
Internalization of GO by undifferentiated Caco-2 cells I. Composed TEM images of undifferentiated Caco-2 cells grown on permeable supports: **A** control cells, **B** after exposure to 20 µg GO1/ml for 24 h. *NC* nucleus, *PCM* polycarbonate (PC) membrane. *Scale bars* 2 µm

**Fig. 4:**
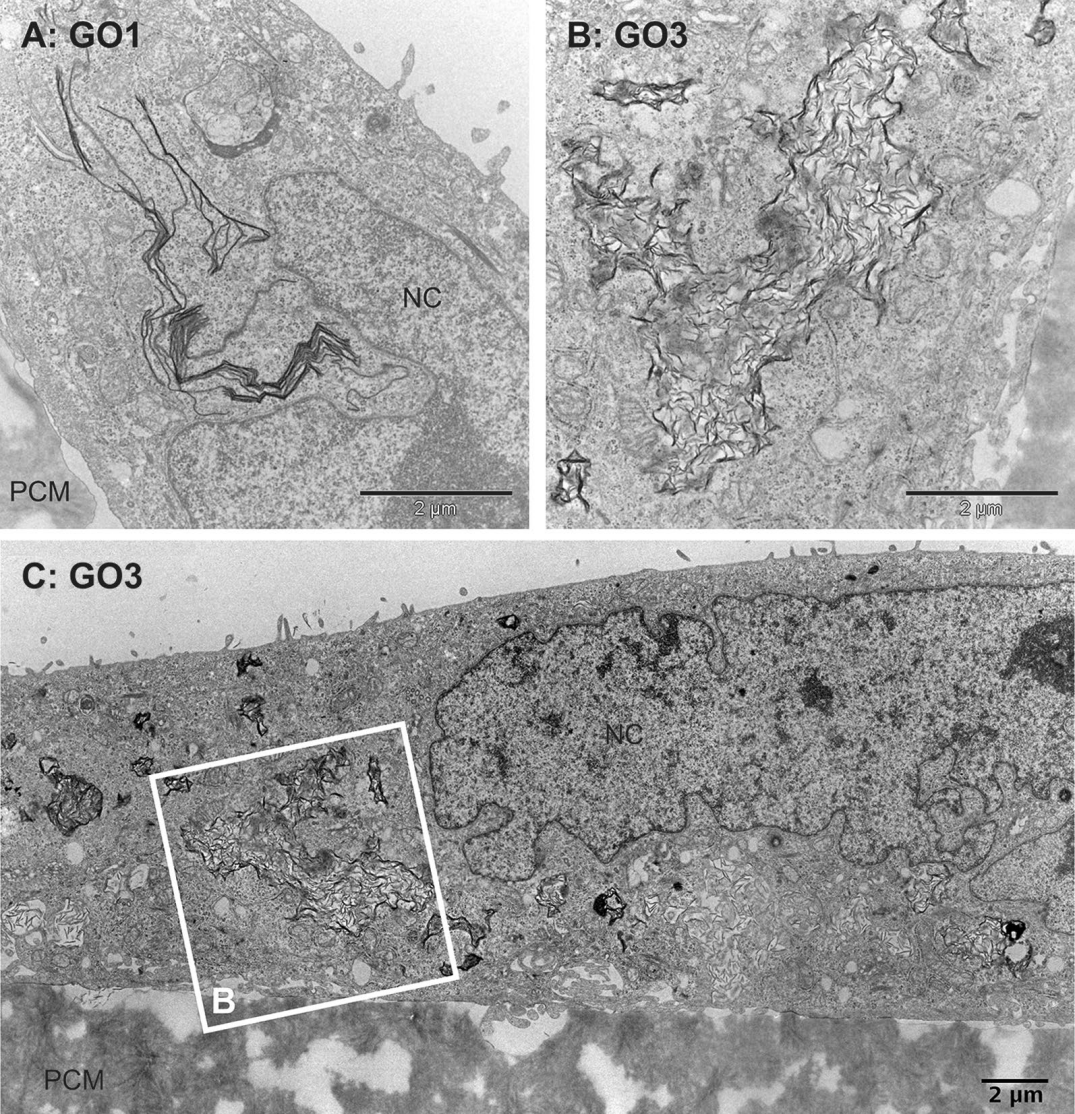
Internalization of GO by undifferentiated Caco-2 cells II. TEM micrographs of undifferentiated Caco-2 cells grown on permeable supports. Caco-2 after exposure to 20 µg/ml GO1 (**A**) or GO3 (**B**, **C**) for 24 h; **C** composed image of TEM micrographs showing parts of a Caco-2 cell with intracellular accumulation of GO3 sheets; **B** higher resolution image of framed area in **C**. *NC* nucleus, *PCM* polycarbonate membrane. *Scale bars* 2 µm

Verification of a possible uptake of GO1 and GO3 by undifferentiated Caco-2 cells was performed by transmission electron microscopy (TEM) analysis. GNP turned out to be a challenging material for TEM analysis. Its rigid structure and general hardness resulted in holes and other artefacts during ultrathin slicing of the samples. This precluded proper analysis and meaningful conclusions. We therefore excluded GNP from TEM analysis. Figure 3A shows a section of neighbouring undifferentiated Caco-2 cells grown in form of islets on polycarbonate membrane (PCM) supports. In contrast to differentiated Caco-2 cells (compare Fig. 8) cells show membrane protrusions instead of microvilli (MV) on the surface. Mitochondria are clearly visible and randomly distributed throughout the cells. Nuclei exhibit deep invaginations forming pockets as well as tunnels. Cellular protrusions can be found on the basal side associated with the PCM pores. Exposure of undifferentiated Caco-2 cells to either 20 µg/ml GO1 or GO3 for 24 h led to a clearly visible internalization of GO sheets. Figure 3B gives an overview on the association of large GO1 sheets with the undifferentiated Caco-2 cells. Unexpectedly uptake was not limited to small GO sheets with lateral dimensions below 1 µm as can be found for GO3 (Fig. 4B, C). Astonishingly, undifferentiated Caco-2 cells were also able to internalize large GO1 sheets with lateral dimensions within the range of 5–10 µm, as can be seen in Figs. 3B and 4A. This is surprising as epithelial cells are not considered as professional phagocytes, not even when of cancerous origin as the Caco-2 cell line. In addition, the TEM images clearly show that uptake of GO sheets was not restricted to a few individual cells but rather the case for a larger number of cells. In the majority of cases internalized GO1 sheets were present in form of structures made of several stacked GO sheets. Interestingly, these structures exhibited folds and sharp bends as shown in Figs. 3B and 4A, indicating a certain flexibility but also rigidity of the stacked material. The stacking of the GO might have occurred before or during cellular uptake, or probably as a combination of both. The sharp-bended structure of the GO1 sheets observed by TEM analysis reflects the structure of the sheets previously observed by SEM analysis, as shown for example in Fig. 2A, B. Also internalized small GO3 sheets were found accumulated throughout the cytoplasm, but with a significantly different structure. Figure 4A, B compare the aspect of GO1 and GO3 within the cell. Our results regarding the uptake of GO3 sheets are in line with other studies investigating the uptake of GO by various cell types. Just recently, uptake of nano-sized GO sheets was reported for HeLa cells as well as for murine macrophages J774.2 [35]. By TEM analysis the authors found significant amounts of GO sheets enclosed in membrane-bound compartments. Both cell-types internalized two different types of nano-sized GO, irrespectively of the lateral size dimension, which was in the range of 89 and 277 nm respectively. Nevertheless, the authors reported a size-dependent effect on the metabolic activity of both cell types, but no elevation of cell death above the normal cell turnover (for GO concentrations up to 100 µg/ml; incubation time 24 and 48 h). In a previous in vitro approach, we did not find a size-dependent effect of GO on the metabolic activity of undifferentiated Caco-2 cells, regardless of the differences in the lateral dimension of GO1, GO3 and other applied GO [20]. Overall, the presented results demonstrate a very strong interaction of the cell membrane with GO leading to the formation of bent GO structures. This strong interaction seems to allow the intestinal epithelial cells to kind of “crumple” and internalize even the large GO1 sheets with lateral dimensions in the size range of whole cells. Despite reports showing the uptake of micro-sized GO sheets by human and murine macrophages [33], which have the function to take up and eliminate foreign materials, to the best of our knowledge there is no study available that previously reported the uptake of large GO sheets with lateral dimensions of up to several tens of micrometres by human epithelial cells.

### Semi-quantification of Caco-2 cells with cell-associated GRM

**Fig. 5:**
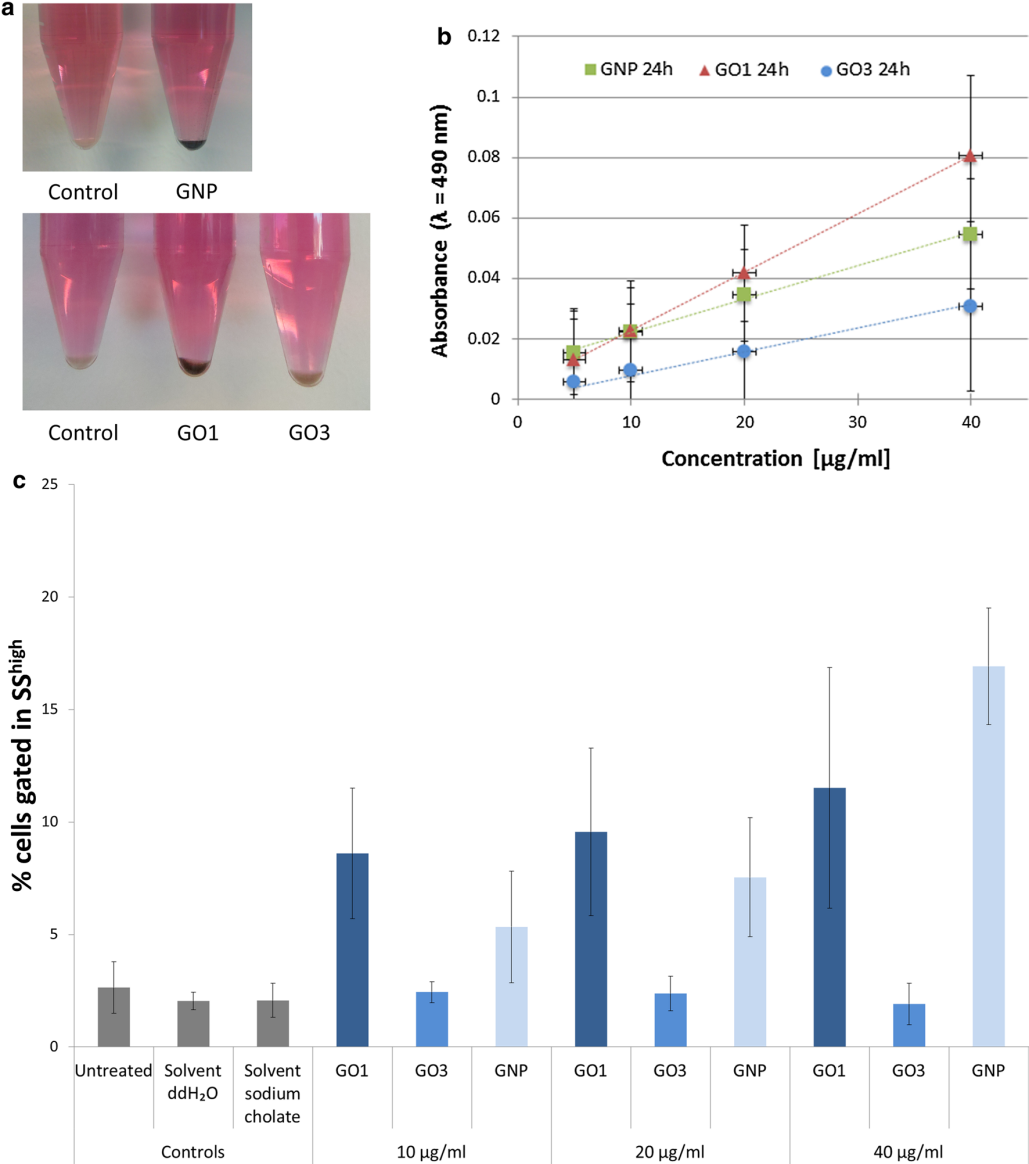
Cell-associated GRM. **a** Undifferentiated Caco-2 cells exposed to GRM for 24 h show GRM-concentration dependent increase in absorbance (absorbance corrected for intrinsic cell absorbance; n = 4 for GNP, n = 6 for GO1 and GO3); **b** images of Caco-2 cell pellets in phenol-red containing cell culture medium including all supplements after exposure to 40 µg GRM/ml for 24 h in comparison to unexposed control cells; **c** flow cytometric analysis (n = 3) shows concentration-dependent increase in % of cells associated with GO1 and GNP. Exposure to GO3 did not lead to a significant increase in the % of cells with cell granularity above the control cells

Undifferentiated Caco-2 cells were treated for 24 h with 40 µg/ml GRM, rinsed, detached by trypsin/EDTA treatment and harvested by centrifugation (Fig. 5a). Dependent on the type of GRM cell pellets appeared either almost black (GNP), dark brown (GO1), or light brown (GO3). This macroscopic and qualitative observation indicates that dependent on the GRM type different amounts of GRM are associated with Caco-2 cells. Absorbance measurements at 490 nm reveal similar results (Fig. 5b; [20]). Treatment of undifferentiated Caco-2 cells with increasing concentrations of the indicated GRM for 24 h results in linearly increasing absorbance values. In this experimental setting GO1 absorbance levels are higher compared to GNP. GO3 treatment only marginally increases absorbance values (Fig. 5b). This effect is most prominent at the highest concentration of 40 µg/ml GRM.

Finally a flow cytometric analysis was carried out to estimate the number of undifferentiated Caco-2 cells associated with GRM (Fig. 5c) as a semi-quantitative measure of interaction and therefore to complement the qualitative TEM- and SEM-data. It has been shown previously that uptake as well as extracellular adhesion of nanoparticles lead to an increase in cell granularity which can be detected by elevated side scatter values [31, 32]. As this method does not allow discrimination between intra- and extracellular GRM, the sum of both is given as “% of cells associated with GRM”. This sub-population was gated in SS^high^ according to the gating strategy presented in Additional file 1: Figure S5. GO1 and GNP treatment of undifferentiated Caco-2 cells for 24 h leads to a concentration-dependent increase in the population of cells associated with GRM (Fig. 5c). At lower concentrations (10 and 20 µg/ml) GO1 treatment leads to more pronounced effects compared to GNP treatment. However, at the highest concentration analysed (40 µg/ml) more cells associate with GNP (17%) compared to GO1 (12%) (Fig. 5c). In contrast in GO3 treated samples side scatter values were not elevated above background levels of untreated and solvent treated control samples at all concentrations analysed. This indicates no or only little uptake and interaction of GO3 with undifferentiated Caco-2 cells. In general the flow cytometric quantification correlates with the macroscopic observations as well as the absorbance measurements: darker cell pellets—higher absorbance values—more cells associated with the respective GRM.

However the increase in side scatter values correlates not only with the amount of a material that is associated to the cell but also with the scattering properties of the respective material itself. As depicted in Additional file 1: Figures S1 and S2, the different GRM (GNP, GO1 and GO3) show distinct scattering properties in differential interference contrast (DIC). While GO1 and GNP show clearly visible surface scattering, GO3 can only be recognized as a change in intracellular architectures of the perinuclear region with low contrast compared to cellular components. These observations may contribute to the differences in absolute side scatter values measured after Caco-2 treatment with different GRM. Nevertheless the applied method allows a relative quantification comparing an untreated sample with corresponding samples treated with different concentrations of the same GRM.

### SEM analysis of the interaction of GO and the cell surface of differentiated Caco-2 cell monolayers

**Fig. 6:**
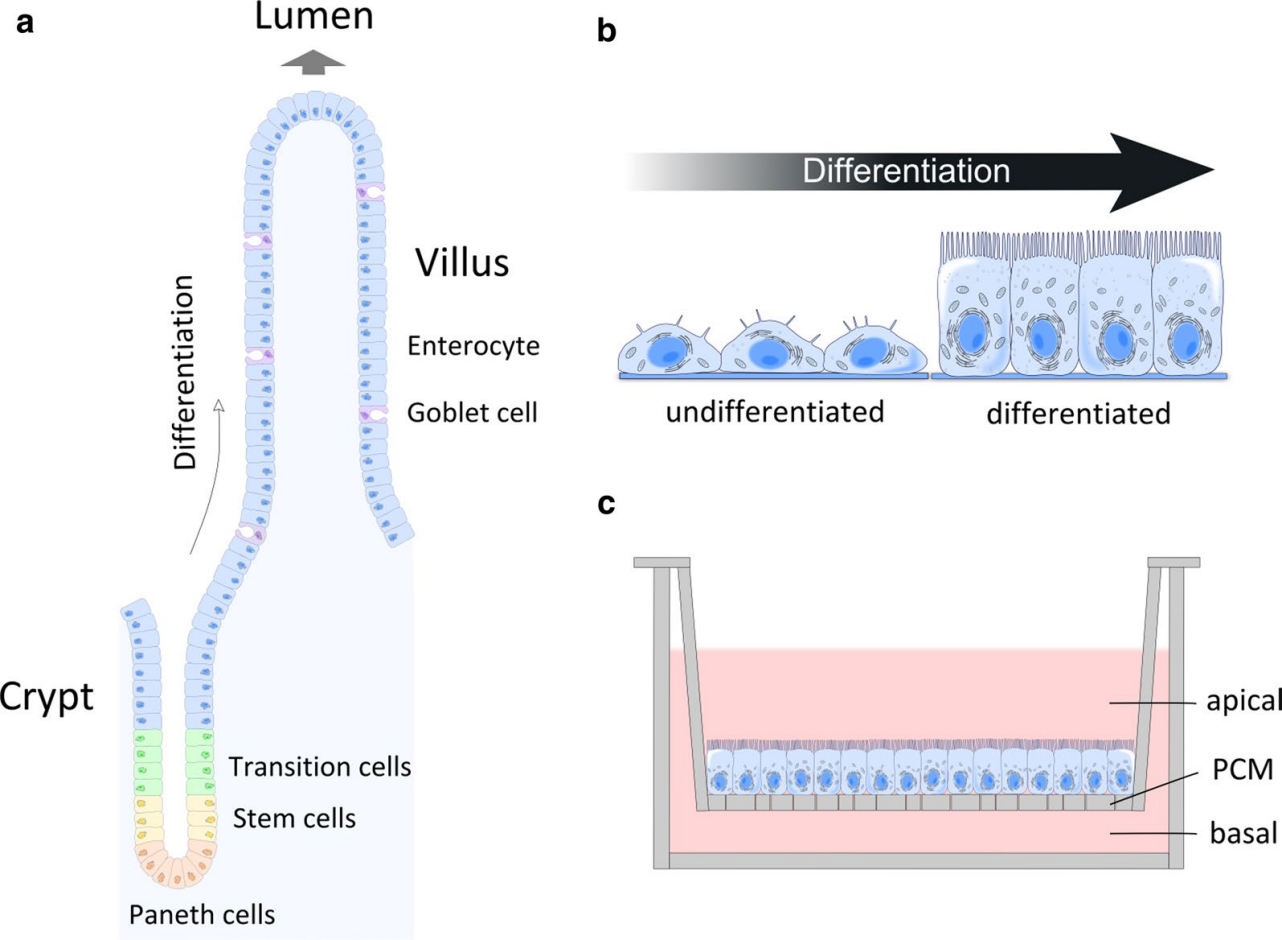
Schematic illustration of the differentiation of intestinal cells. **a** In vivo architecture and organization of the intestinal epithelium (small intestine) based on information given in Refs. [75–77]. At least cells of seven lineages (enterocytes, mucus-secreting goblet cells, enteroendocrine cells, Paneth cells, tuft cells, M-cells, and cup cells) originate from the intestinal stem cells near the crypt base. Enterocytes differentiate during migration from the crypt to the villus tip. **b** Caco-2 cells undergo intense morphological and physiological changes during differentiation. **c** In vitro Transwell^®^ model applied for Caco-2 cell differentiation: diffusion chamber system composed of an apical and basolateral compartment separated by a porous membrane support. Caco-2 cells are grown on the apical side. *PCM* polycarbonate (PC) membrane

**Fig. 7:**
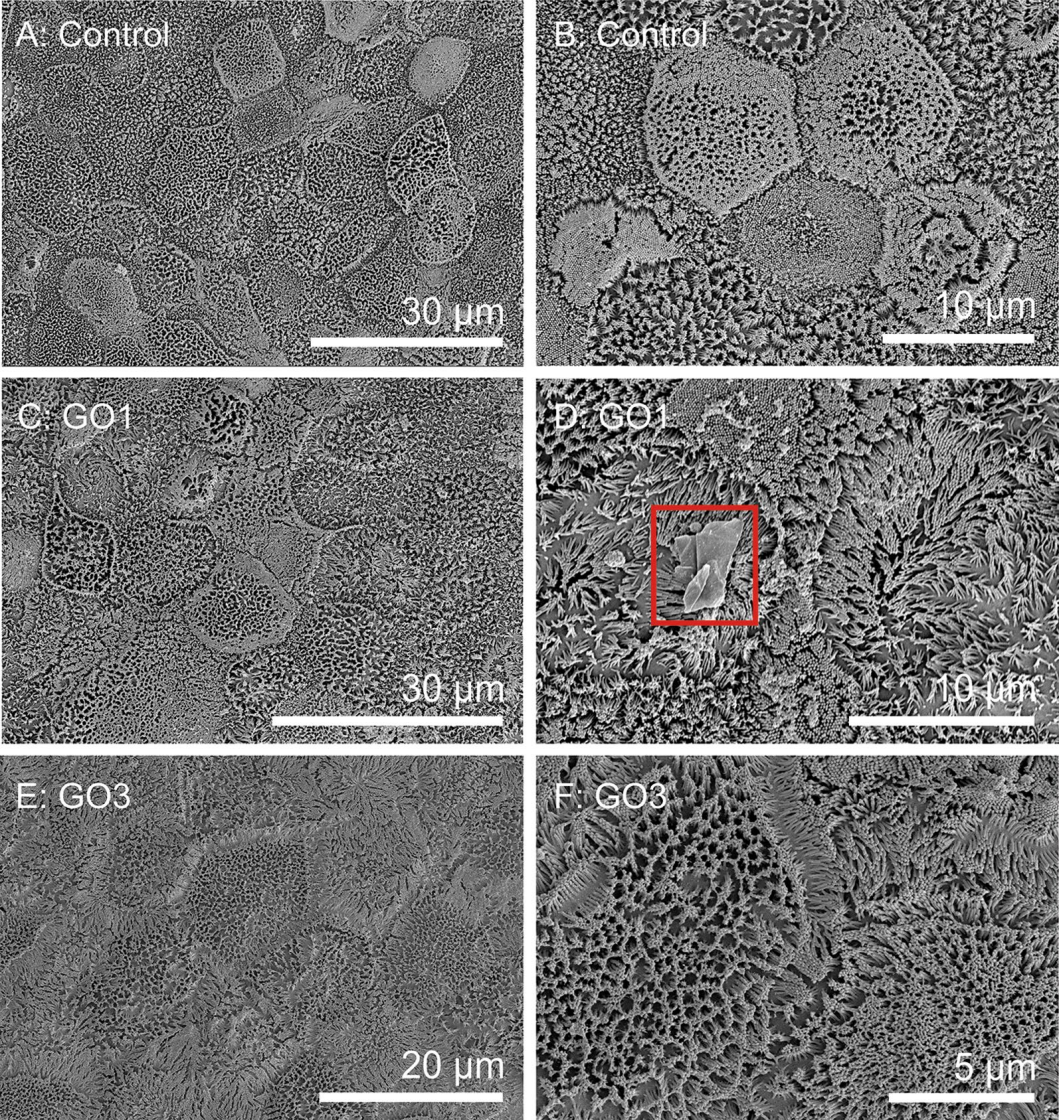
Cell surface morphology of differentiated Caco-2 cells. Scanning electron microscopy (SEM) images of differentiated Caco-2 cells grown on permeable membrane supports. Images show control cells without GO exposure (**A**, **B**) and cells after exposure to 20 μg GO1/ml (**C**, **D**) or 20 μg GO3/ml (**E**, **F**) for 24 h. Only a few GO1 sheets could be identified on the brush border surface (*highlighted* by *red box*), whereas no GO3 sheets could be clearly identified. For further SEM images see Additional File 1: Figure S6

Upon reaching confluency Caco-2 cells stop proliferation and undergo a process of polarization and differentiation to obtain an enterocyte-like phenotype as schematically illustrated in Fig. 6. Differentiation of Caco-2 cells is connected with transcriptome and proteome changes [36–39], as well as an intensive re-modelling of the cell architecture. Characteristic morphological features of differentiated Caco-2 cells are the formation of a dense apical brush border (BB) of close-packed microvilli (MV) (Fig. 7; Additional file 1: Figure S6) and the presence of tight junctions (TJs) (Additional file 1: Figure S7).

To investigate the interaction of GO sheets with the apical surface of the differentiated Caco-2 cell monolayer, cells were exposed to 20 µg/ml GO1 or GO3 for 24 h. Differentiated Caco-2 cells with and without exposure to GO1 showed no significant difference in cell surface morphology. In both cases differentiated Caco-2 cells exhibited a dense BB formed by numerous MV per cell. Density of the brush border varied from cell to cell within the monolayer, which is consistent with Wilson et al. [22] and others. There was no visible difference in average brush border density between the GO-exposed and non-exposed cell layers. In both cases, several cells showed hexagonal arrangement of close-packed MV. Noteworthy, only very few GO sheets could be identified on top of the differentiated GO-exposed Caco-2 cells (Fig. 7; Additional file 1: Figure S6). The surface topography around attached GO sheets did not show any hints towards obvious GO-induced cell surface structure re-arrangements e.g. in form of reduced MV number, length or density. No GO could be found inserted between the MV. GO1 showed weak attachment to the cell monolayer and the majority of sheets were already washed away during the first steps of sample preparation. Residual GO could be rarely found and was often associated to MV bundles with an orientation tilted from the upright position, so that a higher degree of surface interaction with the MV membrane surface was enabled. In contrast to undifferentiated Caco-2 cells differentiated cells showed significantly less cell-associated GO after a similar cell preparation procedure, which indicates differences in the GO-cell surface interaction.

### TEM analysis of GO uptake by differentiated Caco-2 cell monolayers

**Fig. 8:**
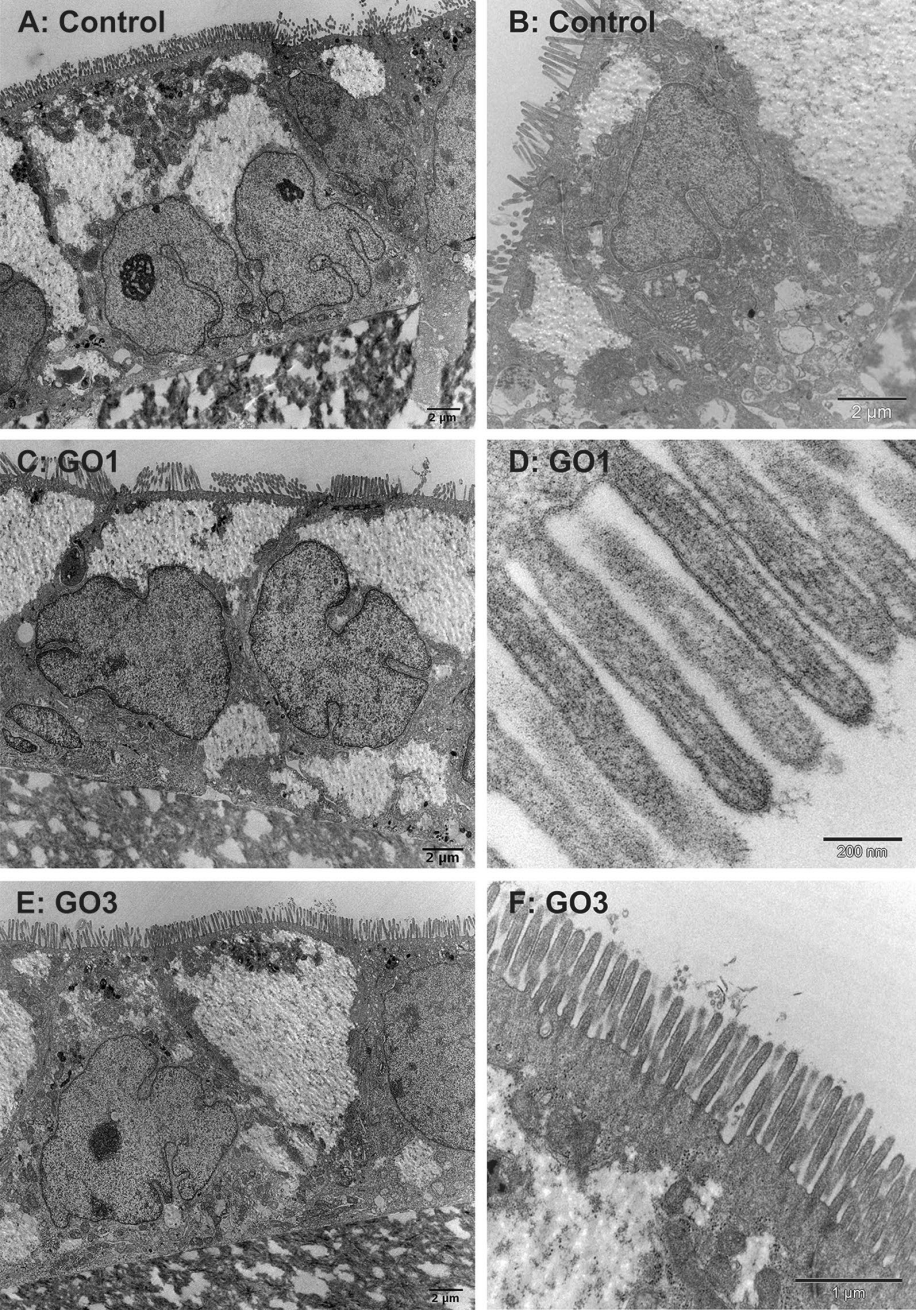
TEM analysis of the interaction of GO and differentiated Caco-2 cells. TEM images of differentiated Caco-2 cells grown on permeable supports: **A**, **B** control cells without GO exposure, **C** Caco-2 cells after exposure to 20 µg/ml GO1 for 24 h. **D**–**F** Caco-2 cell morphology after exposure to 20 µg/ml GO3 for 24 h (**E** polarized cell layer on PC membrane. **D**, **F** Microvilli-arrangement). Neither GO1 nor GO3 sheets could be found closely attached to or internalized by differentiated Caco-2 cells

**Fig. 9:**
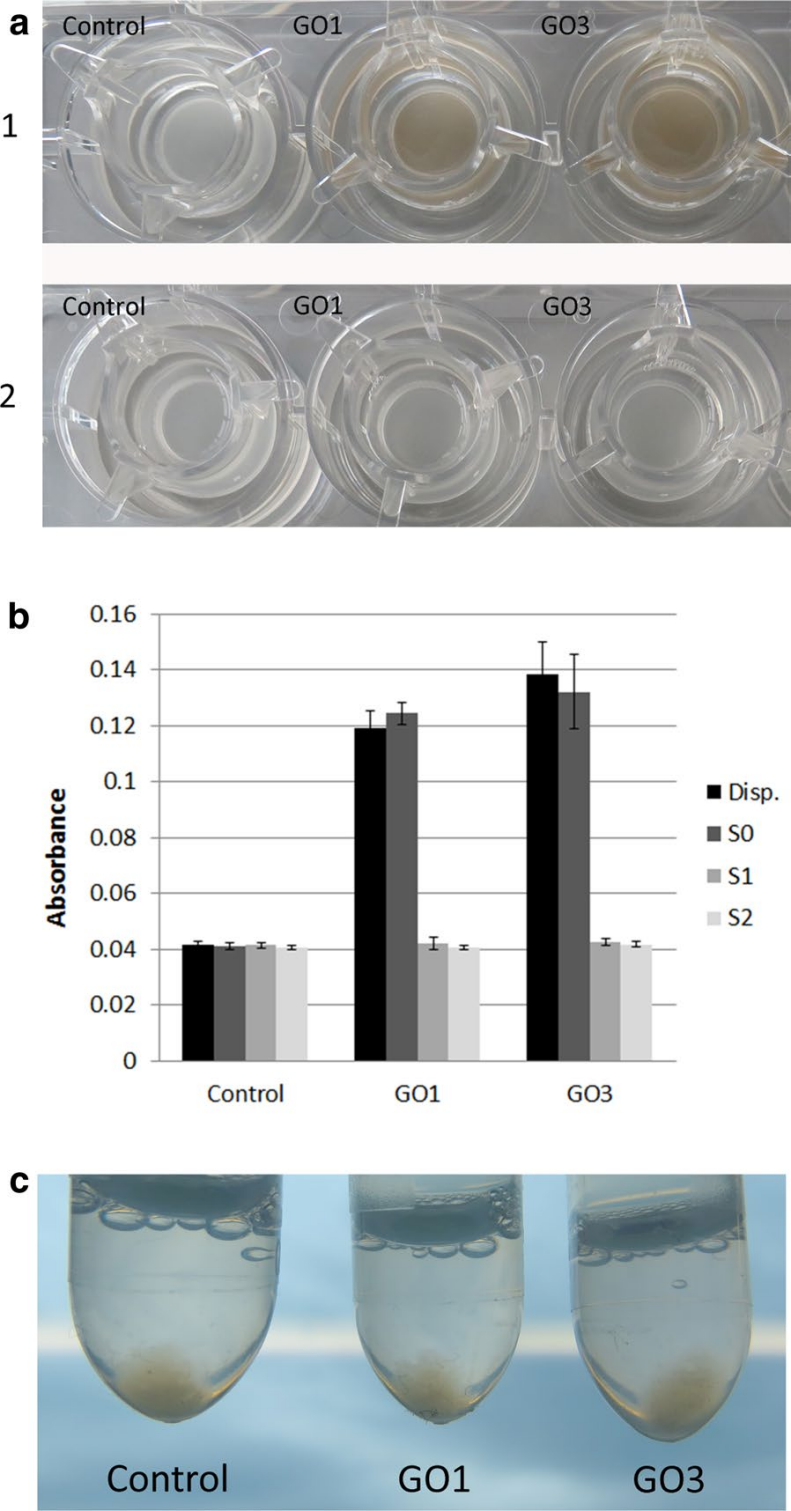
GRM association with differentiated Caco-2. **a** Differentiated Caco-2 cells grown on cell culture inserts (PET membrane). *a1* After exposure to GO1, GO3 or control medium for 24 h; *a2* after exchange of supernatant (S0) by phenol-red free cell culture medium; **b** absorbance spectra of original GO dispersions (40 µg/ml GO) and control medium, as well as of cell culture supernatants after 24 h cell exposure (S0), after one washing step (S1) and two washing steps (S2). **c** Obtained cell pellets of differentiated Caco-2 cells after exposure to GO after two washing steps. No evidence for cell-associated GO given

To investigate whether differentiated Caco-2 cells exhibit similar GO uptake behaviour as undifferentiated Caco-2 cells, differentiated Caco-2 cells were exposed to 20 µg GO1 or GO3 per ml for 24 h (Fig. 8) similar to the undifferentiated Caco-2 cells. Figure 8A, B show differentiated Caco-2 cells without exposure to GO (control cells). The cells exhibit clear polarization with a dense apical BB and TJs. The cell nuclei can be found within the basolateral compartment of the cells and exhibit pockets and tunnels, a finding frequently observed in Caco-2 cells. As already observed by SEM analysis, differentiated Caco-2 cells exposed to GO1 or GO3 for 24 h did not reveal signs of BB disruption, obvious changes in the MV number or length. Figure 8D shows a close-up of adjacent MV of GO1-exposed cells. Parallel alignment of actin filaments within individual MV is clearly visible. MV exhibit a typical length of about 1 µm and width around 0.1 µm as reported by Crawley et al. [40] and others. The presence of TJs further highlights intact cell–cell contacts and does not give hints towards severe effects on the barrier integrity of the Caco-2 cell monolayer. TJs were clearly visible in TEM analysis as well as detected by immunofluorescence labelling of Zonula occludens-1 (ZO-1) proteins (see Additional file 1: Figure S7). Interestingly, no uptake of GO sheets could be observed; neither of large nor of small GO sheets. In case of GO3 some single sheets were found on top of the BB microvilli as shown in Fig. 8F, but no GO sheets could be detected inserted between the MV or within the Caco-2 cells. Based on TEM we cannot totally exclude uptake of unlabelled GO sheets by differentiated Caco-2 cells, but the here presented results clearly show that the incidence and the amount of GO uptake is dramatically reduced for differentiated Caco-2 cells. This conclusion is further confirmed by the following macroscopic observations. In contrast to undifferentiated Caco-2 cells (Fig. 5) differentiated Caco-2 cells treated with GRM exhibit no brown coloration (Fig. 9). After 24 h of treatment with GO1 or GO3 supernatants exhibit the typical brown colour (Fig. 9a1; black (Disp.) and dark grey (S0) bars in Fig. 9b). However, after removal of the supernatant and a single washing step, cells adhering to the permeable support (Fig. 9a2) as well as detached and pelleted cells (Fig. 9c) cannot be distinguished from the untreated control cells. Absorbance values of the original GO dispersions and the corresponding supernatants after 24 h of cell treatment are indistinguishable. In contrast both washing solution (S1 and S2) are at background levels (Fig. 9b). This supports the low adhesion and no or negligible uptake of GO to differentiated Caco-2 cells.

## Discussion

Cellular uptake of nanomaterials is a key process and triggers further biological effects within the cell. We could observe that uptake of label-free GO sheets and other GRM by cells is highly dependent on the cell phenotype. Whereas undifferentiated Caco-2 cells exhibited uptake of small and large GO sheets of several micrometre in size, no GO uptake could be found in differentiated Caco-2 cells. In undifferentiated Caco-2 cells large membrane folds, ruffles and wave-like membrane protrusions engulfing GRM might be seen as hints towards macropinocytosis, an active uptake mechanism, but this still needs to be confirmed. Further evidence for active uptake is reported by Chowdhury et al. who observe uptake of oxidized graphene nanoribbons (GNR) functionalized with PEG-DSPE by HeLa cells. The authors proposed endocytosis as possible uptake mechanism for small GNR aggregates and macropinocytosis for large GNR aggregates [41]. Clear identification of the uptake mechanism is not without difficulties due to the lack of specific markers and inhibitors for macropinocytosis [42]. However, the observed engulfment of GO sheets by undifferentiated Caco-2 cells in our study is not a single event, but was frequently observed for numerous Caco-2 cells in each individual SEM analysis. On the other hand it must be added that just recently, simulations have shown the possibility of passive membrane penetration of graphene and the results were underlined by electron microscopy imaging [15]. Unfortunately, the frequency of such penetration events within the cell culture was not reported by the authors. In contrast, our investigations with undifferentiated Caco-2 cells do not give any hint towards a similar razor blade-like passive entrance of GO through the cell membrane having analysed hundreds of comparable SEM-pictures. However, further experiments would be necessary to elucidate the uptake mechanism(s) of GRM into undifferentiated Caco-2 cells and to exclude any passive way of entrance, even though the results presented herein strongly support an active mechanism of GRM uptake.

To achieve uptake of stacked GO sheets with lateral dimensions of several hundreds of nanometres or more cells have to perform intensive deformation and remodelling of their surface and underlying cytoskeleton. Astonishingly, undifferentiated Caco-2 cells were able to internalize GO sheets with lateral dimensions not far from their own diameter. Our results clearly show that the applied GO sheets are not rigid structures and allow certain deformation of their form. The mechanical properties of GRM have been studied experimentally and by simulation [43–49], but nevertheless remain not fully understood. Monolayer graphene exhibits a high in-plane Young’s modulus [43] but has a high out-of-plane deformability. It has been shown that at the nanometre scale “perfect” graphene is not completely flat. The degree of functionalization and the presence of defects have a significant impact on the materials mechanical behaviour and the formation of extrinsic wrinkles [44, 45]. Stacking of GO sheets can lead to a decrease in the fracture strength and Young’s modulus dependent on the thickness of the GO stack [48]. Chen et al. showed that the bending stiffness of few-layer graphene (2–6 layers) is highly dependent on the thickness and number of graphene layers [50]. As stacking and folding of GRM induces changes in the physical properties the number of GRM layers might have a significant effect on the uptake behaviour of the cells. We made the observation that GO sheets dispersed in cell culture medium undergo different degrees of wrinkling and folding forming simple to highly complex structures. Furthermore, results of our SEM analysis give hints that the here applied GO sheets can be deformed during the cellular uptake process. Undifferentiated Caco-2 cells might be able to generate forces high enough to induce further folding of the sheets. Tymchenko et al. have reported traction forces of adherent cells (endothelial cells and fibroblasts) in the range of tens to hundreds of Nanonewton [51]. It becomes evident that the mechanical properties of GRM are of high relevance and that the lateral dimension of GRM is only one major criterion determining the cellular uptake. As long as the material is deformable, the impact of lateral dimension might be smaller than originally expected and even large GO sheets might be less problematic for cells.

Another important observation is the fact that the differentiated Caco-2 cells exhibit dramatically different uptake efficiency than undifferentiated Caco-2 cells. The reason for this difference is the altered phenotype after polarization and differentiation. As mentioned earlier, upon reaching confluency, Caco-2 cells undergo spontaneous polarization and differentiation, which is connected with progressive changes in cell morphology and multiple biochemical pathways. From a biochemical point of view undifferentiated Caco-2 cells resemble the tumorigenic phenotype, whereas by differentiation they obtain a phenotype similar to enterocytes in healthy tissue [37]. Gene expression profiles of differentiated Caco-2 cells showed similarities with normal human differentiated villus cells and colonic tissue [38, 52]. One possible explanation for the differences in cellular uptake behaviour might be changes in the expression of receptors involved in cellular uptake as was reported for *Yersinia pseudotuberculosis* cell-entry in Caco-2 cells [53]. But to our opinion, the most likely explanation for the differences in GO uptake behaviour between the two phenotypes lies within the architecture of the apical surface. Undifferentiated Caco-2 cells exhibit a few to several protrusions on the cell surface. During differentiation Caco-2 cells undergo extensive remodelling of the apical surface. Microvilli clusters are formed which result in the generation of a dense brush border (BB) as shown in Figs. 7, 8 and Additional file 1: Figure S6, leading to amplification of the surface area of the small intestine. The BB allows an enrichment of membrane-associated enzymes and ion exchangers for nutrient absorption [40, 54], therefore MV are important for the intestinal homeostasis. Perturbation of the BB by loss of MV can lead to malabsorption and diarrhoea. After a close look on the details of the MV architecture and the complexity of the BB, as summarized by Crawley et al. [40], it becomes clear that the BB also serves as a physical barrier. In short, MV are finger-like protrusions (~1 µm in length and ~100 nm in diameter) with a core formed by 20–40 bundled actin filaments necessary for the stability and rigidity of the MV. Each microvillus is anchored into the terminal web which underlies the apical cell surface and promotes the long-term stability of the BB. Neighbouring MV are connected to each other by extracellular inter-microvillar adhesion links at their distal tips [55]. These adhesion links are thought to be important for BB assembly by promoting MV close packing. Interconnection of MV might prevent the occurrence of large gaps within the BB arrangement which could act as protective niches for microbial growth as well as access points for invasion. It is also assumed that they might be involved in obtaining MV with uniform length [40]. In other words, the BB seems to function as kind of mesh or filter which restricts the passage of larger items such as luminal bacteria and micro-sized particles, while allowing passage of molecules and particles small enough to slip through the inter-microvillar spaces. In addition, it could be further speculated that the MV arrangement itself leads to a highly reduced attachment of micrometre sized materials and bacterial cells to the epithelial cell surface. In the 1990s Gebert et al. stated that “the brush border of normal enterocytes is dense and regular, and therefore inhibits the binding of bacteria to larger membrane domains” [56]. Interestingly conversion of Caco-2 cells to M-like cells in the presence of B-lymphocytes results in a reorganization of the BB and uptake of particles and bacteria in contrast to non-converted Caco-2 monolayers [57]. Recently Bennet et al. have shown that the BB can act as an electrostatic barrier repelling intestinal microbes [58]. By generating Caco-2 cells lacking BB formation (microvillus-minus cells, MVM cells), the authors could demonstrate that bacterial adhesion is strongly affected by the presence of MV on the apical surface. Bacteria showed preference for binding to MVM cells rather than to microvilli-possessing Caco-2 cells. Furthermore, the preference for binding was proportional to the zeta-potential of the bacterial particles. In addition, binding of negatively charged poly lactic-co-glycolic acid (PLGA) NPs resembled the binding behaviour of bacteria exhibiting binding preference for MVM cells, whereas positively charged PLGA particles showed preference for the conventional Caco-2 cells. The results clearly show an electrostatic barrier effect of the MV. A similar assumption was made by Vandrangi et al. [59]. A general low adhesion of foreign bodies and bacteria should be beneficial to keep the cell surface “clean”. Adhesion of foreign materials and bacteria would block the surface and should compromise the absorptive function of the cells as well as be beneficial for bacterial invasion. These facts might explain our observation of the low adhesion of GO, which comprises a negative zeta-potential, to the BB of differentiated Caco-2 cells. Therefore the previously described mask-effect, an intimate and parallel alignment of GO sheets to the cell surface was only found for undifferentiated Caco-2 cells, but not for differentiated Caco-2 cells.

The hypothesis that the MV play a decisive role in the barrier function is further strengthened by the fact that M-cells, which are specialized for sampling luminal microbial particles and food antigens, lack a BB on their apical surface [60, 61]. Gebert et al. assumed that “the irregular or even flat surface of M cells could facilitate the association of potential antigens with the M cell surface.” [56]. Just recently Schimpel et al. have shown that M-cells exhibit a 1.7-fold higher cell elasticity compared to Caco-2 cells with BB, as well as higher adhesion forces to the applied AFM tip [62]. The authors assumed that the sparse arrangement of MV and the increased elasticity are responsible for the high endocytic activity of M-cells. These results further highlight the importance of cell surface topography and deformability for cellular uptake behaviour. Therefore, M-cells would be a very interesting model in regard of GRM uptake. Furthermore, it was shown that entero-pathogenic *Escherichia coli* (EPEC) and *Salmonella typhimurium* have evolved mechanisms to induce re-modelling of the apical surface of human enterocytes leading to disruption of the BB and adhesion of the bacteria [63, 64].

Our results are further in line with an in vitro study performed by Clark et al. which assessed the interactions of pristine and oxidized multi-walled carbon nanotubes (MWCNT; 10–20 nm in diameter, 200–500 nm length) and differentiated Caco-2 cells. The authors found no evidence for an uptake of neither pristine nor oxidized MWCNT by differentiated Caco-2 cells [65].

Uptake of spherical NPs with 50 nm in size, but not for 100 nm particles and larger, has been demonstrated for differentiated Caco-2 cells [66]. Similarly NPs smaller than 40 nm were found in enterocytes of mouse small intestine after per-oral and intraluminal administration, whereas NP larger than 100 nm were not found [67]. Janer et al. reported no uptake of TiO_2_ NP (primary particle size ~18 nm) by differentiated Caco-2 cells in vitro as well as no detectable TiO_2_ NP in sections of the small intestine of exposed rats in vivo [68]. Peuschel et al. have shown uptake of amino- and carboxyl-functionalized CdSe/ZnS quantum dots of around 14 nm in size by undifferentiated Caco-2 cells, but not by differentiated Caco-2 cells [69]. Comparable results were obtained for 26 and 100 nm TiO_2_ NPs and their aggregates by Song et al. [70].

Taken together, the enterocyte architecture is an extremely efficient barrier not only for micro-sized but also for nano-sized foreign materials. The results indicate that larger particles and NP-aggregates suffer from sterical hindrance and are therefore excluded, whereas particles with sizes smaller than the distance between adjacent MV bear the potential to pass through the BB, but are not necessarily taken up by the cells. Uptake of GRM and other 2D materials, even when consisting of only one or few layers, so with thickness in the range of 1–10 nm, but with lateral dimensions in the upper nanometre or micrometre range, seems to be very unlikely for differentiated Caco-2 cells. Such materials might bear the potential for transcellular passage of the enterocytes only in case of perturbation of the BB and terminal web.

It has to be noted that one limitation of the here applied Caco-2 cell model is that it does not consider the mucus layer covering and protecting the cells in the intestinal tract. Recently, Sinnecker et al. have shown in an ex vivo model of the rat intestine that the mucus layer is a critical barrier to overcome. Model polystyrene NPs were trapped within the mucus and no absorption of particles could be found in the applied experimental set-up [71]. Therefore the here presented experimental conditions with differentiated Caco-2 cells resembles the situation where the mucus barrier would be disturbed and a direct contact of the GRM with the epithelial cells is possible. Based on these facts and the results obtained by our study, the potential of GRM with lateral dimensions of several hundreds of nanometres or more to pass through the healthy intestinal barrier by transcytosis of enterocytes seems to be extremely unlikely. Nevertheless, even if enterocytes are the most abundant cell type of the intestinal barrier, the passage and uptake of GRM by other cell types found in the intestinal mucosa such as M-cells, goblet cells, enteroendocrine cells, Paneth cells as well as stem cells cannot be excluded and has to be determined in future investigations. Furthermore 2D- or 3D models composed of different cell types are needed to mimic the complexity of the intestinal barrier and to improve in vitro-in vivo correlation [26, 72, 73]. Models including intestinal microbes or considering the mechanical forces by peristalsis can further increase the physiological relevance. Disease models can help to understand nano- and microparticle translocation across the intestinal barrier under inflammatory conditions [66, 74].

## Conclusions

We can conclude that the GRM uptake behaviour of Caco-2 cells is highly dependent on the phenotype given by the cell differentiation status. Whereas undifferentiated Caco-2 cells were able to internalize label-free GO sheets probably by macropinocytosis, no GRM uptake could be found for differentiated Caco-2 cells. We can further conclude that the mechanical properties of the GRM, such as deformability of the materials, seems to be an important factor for cellular uptake by undifferentiated Caco-2 cells allowing internalization of even large GO sheets as large as 10 µm.

Our results highlight the importance of using appropriate cell culture models. Undifferentiated Caco-2 cells can be applied in screening approaches to identify hazardous nanomaterials. However the model’s predictability is limited, due to significant differences in comparison to human enterocytes in vivo. During differentiation Caco-2 cells undergo intense phenotypic changes leading to an enterocyte-like morphology similar to enterocytes in the human body. The BB alters the cell surface properties such as the topography and leads to repellent effects which apparently results in a low adhesion of GO sheets and lack of uptake. Passage of GO through healthy enterocytes is therefore expected to be extremely unlikely.

## Additional file

**Additional file 1: Figure S1.** Differential interference contrast (DIC) images of non-confluent Caco-2 cells grown on glass cover slips. Cells were exposed to either 20 μg/ml GO1, GO3 or GNP for 24 hours. GRM-exposed cells showed no morphological differences in comparison to the unexposed control cells. Accumulation of dark material in the perinuclear region gives hints towards uptake of GO3 by non-confluent Caco-2 cells. Microscopy analysis was performed with an Axio ImagerZ.1 microscope (Zeiss, Oberkochen, Germany). **Figure S2.** Fluorescence microscopy images (overlays) of non-confluent Caco-2 cells. Cells were exposed for 24 hours to 20 μg/ml GO1, GO3 or GNP respectively. Control cells not exposed to GO were run in parallel. Cell nuclei were labelled with DAPI (blue; λex = 335-383 nm, λem = 420-470 nm). Actin-network was labelled with Phalloidin-Alexa Fluor® 488 (green; λex = 455-495 nm, λem = 505-555 nm). GRM is visible by transmitted differential interference contrast (TL DIC) microscopy. Microscopy analysis was performed with an Axio ImagerZ.1 microscope (Zeiss, Oberkochen, Germany). **Figure S3.** SEM images of non-confluent Caco-2 cells after exposure to GO1 or GO3 for 24 hours. Cells were exposed to 40 μg GO1/ml (top, left) or 20 μg GO1/ml (top, right). GO1 sheets exhibited either highly crumpled morphology especially at the cell-substrate border or were aligned parallel to the cell surface. GO3 was applied in a concentration of 40 μg GO3/ml. GO3 is visible in form of mat-like agglomerates of folded and wrinkled sheets both on the substrate and cell surface (bottom, right). Formation of circular wave-like protrusions on the surface of GO3-exposed cells give hints towards the possible uptake mechanism macropinocytosis (bottom, left). **Figure S4.** Interaction of GNP and the surface of non-confluent Caco-2 cells. SEM images of cells after exposure to 20 μg GNP/ml for 24 h. Most of the shown GNP aggregates were found on the cell surface near the edges of Caco-2 islets and were associated with membrane protrusions. Bottom images: GNP aggregate is exemplarily displayed in purple to facilitate identification. **Figure S5.** FACS analysis. Scatter plots of non-confluent Caco-2 cells after 24 h exposure to GRM. **Figure S6.** Cell surface morphology of differentiated Caco-2 cells. SEM images of control cells without GO exposure and cells after exposure to 20 μg GO1/ml for 24 h. Only a few GO1 sheets could be identified on top of the brush border (highlighted by red boxes). **Figure S7.** Fluorescence microscopy images of differentiated Caco-2 cell monolayer. Control cells without GO exposure and Caco-2 cells after exposure to GO; cells were exposed for 24 hours to 20 μg GO1/ml or 20 μg GO3/ml respectively. Channel 1 (Ch1; λex = 335-383 nm, λem = 420-470 nm) shows cell nuclei labelled with DAPI (blue). Channel 2 (Ch2; λex = 455-495 nm, λem = 505-555 nm) shows the presence of tight junctions by labelling with ZO-1 mouse monoclonal antibody-Alexa Fluor® 488 (green). Channel 3 (Ch3; λex = 538-562 nm, λem = 570-640 nm) shows the actin-network labelled with Phalloidin-Alexa Fluor® 546.

## References

[1] Ferrari AC, Bonaccorso F, Fal’ko V, Novoselov KS, Roche S, Boggild P, Borini S, Koppens FH, Palermo V, Pugno N (2015). Science and technology roadmap for graphene, related two-dimensional crystals, and hybrid systems. Nanoscale.

[2] Novoselov KS, Fal′ko VI, Colombo L, Gellert PR, Schwab MG, Kim K (2012). A roadmap for graphene. Nature.

[3] Samorì P, Kinloch IA, Feng X, Palermo V (2015). Graphene-based nanocomposites for structural and functional applications: using 2-dimensional materials in a 3-dimensional world. 2D Mater.

[4] Hong BJ, Compton OC, An Z, Eryazici I, Nguyen ST (2012). Successful stabilization of graphene oxide in electrolyte solutions: enhancement of biofunctionalization and cellular uptake. ACS Nano.

[5] Liu Z, Robinson JT, Sun X, Dai H (2008). PEGylated nanographene oxide for delivery of water-insoluble cancer drugs. J Am Chem Soc.

[6] Kim H, Lee D, Kim J, Kim T-i, Kim WJ (2013). Photothermally triggered cytosolic drug delivery via endosome disruption using a functionalized reduced graphene oxide. ACS Nano.

[7] Krug HF, Wick P (2011). Nanotoxicology: an interdisciplinary challenge. Angew Chem Int Ed Engl.

[8] Yang K, Zhang S, Zhang G, Sun X, Lee ST, Liu Z (2010). Graphene in mice: ultrahigh in vivo tumor uptake and efficient photothermal therapy. Nano Lett.

[9] Sanchez VC, Jachak A, Hurt RH, Kane AB (2012). Biological interactions of graphene-family nanomaterials: an interdisciplinary review. Chem Res Toxicol.

[10] Seabra AB, Paula AJ, de Lima R, Alves OL, Duran N (2014). Nanotoxicity of graphene and graphene oxide. Chem Res Toxicol.

[11] Orecchioni M, Bedognetti D, Sgarrella F, Marincola FM, Bianco A, Delogu LG (2014). Impact of carbon nanotubes and graphene on immune cells. J Transl Med.

[12] Yue H, Wei W, Yue Z, Wang B, Luo N, Gao Y, Ma D, Ma G, Su Z (2012). The role of the lateral dimension of graphene oxide in the regulation of cellular responses. Biomaterials.

[13] Zhang S, Yang K, Feng L, Liu Z (2011). In vitro and in vivo behaviors of dextran functionalized graphene. Carbon.

[14] Mu Q, Su G, Li L, Gilbertson BO, Yu LH, Zhang Q, Sun YP, Yan B (2012). Size-dependent cell uptake of protein-coated graphene oxide nanosheets. ACS Appl Mater Interfaces.

[15] Li Y, Yuan H, von dem Bussche A, Creighton M, Hurt RH, Kane AB, Gao H (2013). Graphene microsheets enter cells through spontaneous membrane penetration at edge asperities and corner sites. Proc Natl Acad Sci USA.

[16] Krug HF (2014). Nanosafety research—are we on the right track?. Angew Chem Int Ed Engl.

[17] Pierleoni D, Xia ZY, Christian M, Ligi S, Minelli M, Morandi V, Doghieri F, Palermo V (2016). Graphene-based coatings on polymer films for gas barrier applications. Carbon.

[18] Casiraghi C, Robertson J, Ferrari AC (2007). Diamond-like carbon for data and beer storage. Mater Today.

[19] De Marzi L, Ottaviano L, Perrozzi F, Nardone M, Santucci S, De Lapuente J, Borras M, Treossi E, Palermo V, Poma A (2014). Flake size-dependent cyto and genotoxic evaluation of graphene oxide on in vitro A549, Caco_2_ and vero cell lines. J Biol Regul Homeost Agents.

[20] Kucki M, Rupper P, Sarrieu C, Melucci M, Treossi E, Schwarz A, León V, Kraegeloh A, Flahaut E, Vázquez E (2016). Interaction of graphene-related materials with human intestinal cells: an in vitro approach. Nanoscale.

[21] Nguyen TH, Lin M, Mustapha A (2015). Toxicity of graphene oxide on intestinal bacteria and Caco-2 cells. J Food Prot.

[22] Wilson G, Hassan IF, Dix CJ, Williamson I, Shah R, Mackay M, Artursson P (1990). Transport and permeability properties of human Caco-2 cells: an in vitro model of the intestinal epithelial cell barrier. J Control Release.

[23] Artursson P (1990). Epithelial transport of drugs in cell culture. I: a model for studying the passive diffusion of drugs over intestinal absorbtive (Caco-2) cells. J Pharm Sci.

[24] Artursson P, Palm K, Luthman K (2001). Caco-2 monolayers in experimental and theoretical predictions of drug transport. Adv Drug Deliv Rev.

[25] Hidalgo IJ, Raub TJ, Borchardt RT (1989). Characterization of the human colon carcinoma cell line (Caco-2) as a model system for intestinal epithelial permeability. Gastroenterology.

[26] Pereira C, Costa J, Sarmento B, Araújo F, Sarmento B (2016). Cell-based in vitro models for intestinal permeability studies. Concepts and models for drug permeability studies: cell and tissue based in vitro culture models.

[27] Kasper JY, Hermanns MI, Cavelius C, Kraegeloh A, Jung T, Danzebrink R, Unger RE, Kirkpatrick CJ (2016). The role of the intestinal microvasculature in inflammatory bowel disease: studies with a modified Caco-2 model including endothelial cells resembling the intestinal barrier in vitro. Int J Nanomed.

[28] Walter E, Janich S, Roessler BJ, Hilfinger JM, Amidon GL (1996). HT29-MTX/Caco-2 cocultures as an in vitro model for the intestinal epithelium. In vitro–in vivo correlation with permeability data from rats and humans. J Pharm Sci.

[29] Wikman-Larhed A, Artursson P (1995). Co-cultures of human intestinal goblet (HT29-H) and absorptive (Caco-2) cells for studies of drug and peptide absorption. Eur J Pharm Sci.

[30] Liscio A, Veronese GP, Treossi E, Suriano F, Rossella F, Bellani V, Rizzoli R, Samorì P, Palermo V (2011). Charge transport in graphene–polythiophene blends as studied by Kelvin Probe Force Microscopy and transistor characterization. J Mater Chem.

[31] Zucker RM, Massaro EJ, Sanders KM, Degn LL, Boyes WK (2010). Detection of TiO_2_ nanoparticles in cells by flow cytometry. Cytom A.

[32] Ulrich S, Hirsch C, Diener L, Wick P, Rossi RM, Bannwarth M, Boesel LF (2016). Preparation of ellipsoid-shaped supraparticles with modular compositions and investigation of shapedependent cell-uptake. RSC Adv.

[33] Russier J, Treossi E, Scarsi A, Perrozzi F, Dumortier H, Ottaviano L, Meneghetti M, Palermo V, Bianco A (2013). Evidencing the mask effect of graphene oxide: a comparative study on primary human and murine phagocytic cells. Nanoscale.

[34] Palermo V, Kinloch IA, Ligi S, Pugno NM (2016). Nanoscale mechanics of graphene and graphene oxide in composites: a scientific and technological perspective. Adv Mater.

[35] Mendes RG, Koch B, Bachmatiuk A, Ma X, Sanchez S, Damm C, Schmidt OG, Gemming T, Eckert J, Rümmeli MH (2015). A size dependent evaluation of the cytotoxicity and uptake of nanographene oxide. J Mater Chem B.

[36] Tadjali M, Seidelin JB, Olsen J, Troelsen JT (2002). Transcriptome changes during intestinal cell differentiation. Biochem Biophys Acta.

[37] Stierum R, Gaspari M, Dommels Y, Ouatas T, Pluk H, Jespersen S, Vogels J, Verhoeckx K, Groten J, Ommen B (2003). Proteome analysis reveals novel proteins associated with proliferation and differentiation of the colorectal cancer cell line Caco-2. Biochim Biophys Acta (BBA)–Proteins Proteom.

[38] Saaf AM, Halbleib JM, Chen X, Yuen ST, Leung SY, Nelson WJ, Brown PO (2007). Parallels between global transcriptional programs of polarizing Caco-2 intestinal epithelial cells in vitro and gene expression programs in normal colon and colon cancer. Mol Biol Cell.

[39] Halbleib JM, Saaf AM, Brown PO, Nelson WJ (2007). Transcriptional modulation of genes encoding structural characteristics of differentiating enterocytes during development of a polarized epithelium in vitro. Mol Biol Cell.

[40] Crawley SW, Mooseker MS, Tyska MJ (2014). Shaping the intestinal brush border. J Cell Biol.

[41] Mullick Chowdhury S, Lalwani G, Zhang K, Yang JY, Neville K, Sitharaman B (2013). Cell specific cytotoxicity and uptake of graphene nanoribbons. Biomaterials.

[42] Kerr MC, Teasdale RD (2009). Defining macropinocytosis. Traffic.

[43] Lee C, Wei X, Kysar JW, Hone J (2008). Measurement of the elastic properties and intrinsic strength of monolayer graphene. Science.

[44] Gómez-Navarro C, Burghard M, Kern K (2008). Elastic properties of chemically derived single graphene sheets. Nano Lett.

[45] Zheng Q, Geng Y, Wang S, Li Z, Kim J-K (2010). Effects of functional groups on the mechanical and wrinkling properties of graphene sheets. Carbon.

[46] Gomez-Navarro C, Meyer JC, Sundaram RS, Chuvilin A, Kurasch S, Burghard M, Kern K, Kaiser U (2010). Atomic structure of reduced graphene oxide. Nano Lett.

[47] Liu L, Zhang J, Zhao J, Liu F (2012). Mechanical properties of graphene oxides. Nanoscale.

[48] Gong T, Lam DV, Liu R, Won S, Hwangbo Y, Kwon S, Kim J, Sun K, Kim J-H, Lee S-M, Lee C (2015). Thickness dependence of the mechanical properties of free-standing graphene oxide papers. Adv Func Mater.

[49] Meyer JC, Geim AK, Katsnelson MI, Novoselov KS, Booth TJ, Roth S (2007). The structure of suspended graphene sheets. Nature.

[50] Chen X, Yi C, Ke C (2015). Bending stiffness and interlayer shear modulus of few-layer graphene. Appl Phys Lett.

[51] Tymchenko N, Wallentin J, Petronis S, Bjursten LM, Kasemo B, Gold J (2007). A novel cell force sensor for quantification of traction during cell spreading and contact guidance. Biophys J.

[52] Tremblay E, Auclair J, Delvin E, Levy E, Menard D, Pshezhetsky AV, Rivard N, Seidman EG, Sinnett D, Vachon PH, Beaulieu JF (2006). Gene expression profiles of normal proliferating and differentiating human intestinal epithelial cells: a comparison with the Caco-2 cell model. J Cell Biochem.

[53] Coconnier M-H, Bernet-Camard M-F, Servin AL (1994). How intestinal epithelial cell differentiation inhibits the cell-entry of *Yersinia pseudotuberculosis* in colon carcinoma Caco-2 cell line in culture. Differentiation.

[54] Delacour D, Salomon J, Robine S, Louvard D (2016). Plasticity of the brush border—the yin and yang of intestinal homeostasis. Nat Rev Gastroenterol Hepatol.

[55] Crawley SW, Shifrin DA, Grega-Larson NE, McConnell RE, Benesh AE, Mao S, Zheng Y, Zheng QY, Nam KT, Millis BA (2014). Intestinal brush border assembly driven by protocadherin-based intermicrovillar adhesion. Cell.

[56] Gebert A, Rothkötter H-J, Pabst R (1996). M cells in Peyer’s patches of the intestine. Int Rev Cytol.

[57] Kernéis S, Bogdanova A, Kraehenbuhl J-P, Pringault E (1997). Conversion by Peyer’s patch lymphocytes of human enterocytes into M cells that transport bacteria. Science.

[58] Bennett KM, Walker SL, Lo DD (2014). Epithelial microvilli establish an electrostatic barrier to microbial adhesion. Infect Immun.

[59] Vandrangi P, Lo DD, Kozaka R, Ozaki N, Carvajal N, Rodgers VGJ (2013). Electrostatic properties of confluent Caco-2 cell layer correlates to their microvilli growth and determines underlying transcellular flow. Biotechnol Bioeng.

[60] Neutra MR, Kraehenbuhl J-P (1993). The role of transepithelial transport by M cells in microbial invasion and host defense. J Cell Sci.

[61] Neutra MR, Pringault E, Kraehenbuhl J-P (1996). Antigen sampling across epithelial barriers and induction of mucosal immune responses. Annu Rev Immunol.

[62] Schimpel C, Werzer O, Frohlich E, Leitinger G, Absenger-Novak M, Teubl B, Zimmer A, Roblegg E (2015). Atomic force microscopy as analytical tool to study physico-mechanical properties of intestinal cells. Beilstein J Nanotechnol.

[63] Knutton S, LLoyd DR, McNeish A (1987). Adhesion of enteropathogenic *Escherichia coli* to human intestinal enterocytes and cultured human intestinal Mucosa. Infect Immun.

[64] Goosney DL, Knoechel DG, Finlay BB (1999). Enteropathogenic *E. coli*, *Salmonella*, and *shigella*: masters of host cell cytoskeletal exploitation. Emerg Infect Dis.

[65] Clark KA, O’Driscoll C, Cooke CA, Smith BA, Wepasnick K, Fairbrother DH, Lees PS, Bressler JP (2012). Evaluation of the interactions between multiwalled carbon nanotubes and Caco-2 cells. J Toxicol Environ Health A.

[66] Leonard F, Collnot E-M, Lehr C-M (2010). A three-dimensional coculture of enterocytes, monocytes and dendritic cells to model inflamed intestinal mucosa in vitro. Mol Pharm.

[67] Howe SE, Lickteig DJ, Plunkett KN, Ryerse JS, Konjufca V (2014). The uptake of soluble and particulate antigens by epithelial cells in the mouse small intestine. PLoS ONE.

[68] Janer G, del Molino ME, Fernandez-Rosas E, Fernandez A, Vazquez-Campos S (2014). Cell uptake and oral absorption of titanium dioxide nanoparticles. Toxicol Lett.

[69] Peuschel H, Ruckelshausen T, Kiefer S, Silina Y, Kraegeloh A (2016). Penetration of CdSe/ZnS quantum dots into differentiated vs undifferentiated Caco-2 cells. J Nanobiotechnol.

[70] Song ZM, Chen N, Liu JH, Tang H, Deng X, Xi WS, Han K, Cao A, Liu Y, Wang H (2015). Biological effect of food additive titanium dioxide nanoparticles on intestine: an in vitro study. J Appl Toxicol.

[71] Sinnecker H, Krause T, Koelling S, Lautenschlager I, Frey A (2014). The gut wall provides an effective barrier against nanoparticle uptake. Beilstein J Nanotechnol.

[72] Yu J, Peng S, Luo D, March JC (2012). In vitro 3D human small intestinal villous model for drug permeability determination. Biotechnol Bioeng.

[73] Frohlich E, Roblegg E (2016). Oral uptake of nanoparticles: human relevance and the role of in vitro systems. Arch Toxicol.

[74] Susewind J, de Souza Carvalho-Wodarz C, Repnik U, Collnot EM, Schneider-Daum N, Griffiths GW, Lehr CM (2016). A 3D co-culture of three human cell lines to model the inflamed intestinal mucosa for safety testing of nanomaterials. Nanotoxicology.

[75] Potten CS, Loeffler M (1990). Stem cells: attributes, cycles, spirals, pitfalls and uncertainties. Lessons for and from the Crypt. Development.

[76] Moore KA, Lemischka IR (2006). Stem cells and their niches. Science.

[77] Gerbe F, Legraverend C, Jay P (2012). The intestinal epithelium tuft cells: specification and function. Cell Mol Life Sci.

