# Supplementary material for: Uptake of label-free graphene oxide by Caco-2 cells is dependent on the cell differentiation status
Source: J Nanobiotechnology. 2017 Jun 21;15:46. doi: 10.1186/s12951-017-0280-7 (PMC5480125; doi:10.1186/s12951-017-0280-7)

**Additional File 1**

**Uptake of label-free graphene oxide by Caco-2 cells is dependent on the cell differentiation status**

*Melanie Kucki,a* Liliane Diener,a Nils Bohmer,a Cordula Hirsch,a Harald F. Krug,b Vincenzo Palermo,c and Peter Wick,a**

a Laboratory for Particles-Biology Interactions, Empa - Swiss Federal Laboratories for Materials Science and Technology, Lerchenfeldstrasse 5, CH-9014 St. Gallen, Switzerland

b International Research Cooperations Manager, Empa - Swiss Federal Laboratories for Materials Science and Technology, Lerchenfeldstrasse 5, CH-9014 St. Gallen, Switzerland

c Istituto per la Sintesi Organica e la Fotoreattività, Consiglio Nazionale delle Richerche (CNR), Via P. Gobetti 101, 40129 Bologna, Italy

*Corresponding authors: [melanie.kucki@empa.ch,](mailto:melanie.kucki@empa.ch) [peter.wick@empa.ch](mailto:peter.wick@empa.ch)

**Figure S1.** Differential interference contrast (DIC) images of non-confluent Caco-2 cells grown on

glass cover slips. Cells were exposed to either 20 µg/ml GO1, GO3 or GNP for 24 hours. GRM- exposed cells showed no morphological differences in comparison to the unexposed control cells. Accumulation of dark material in the perinuclear region gives hints towards uptake of GO3 by non- confluent Caco-2 cells. Microscopy analysis was performed with an Axio ImagerZ.1 microscope (Zeiss, Oberkochen, Germany).


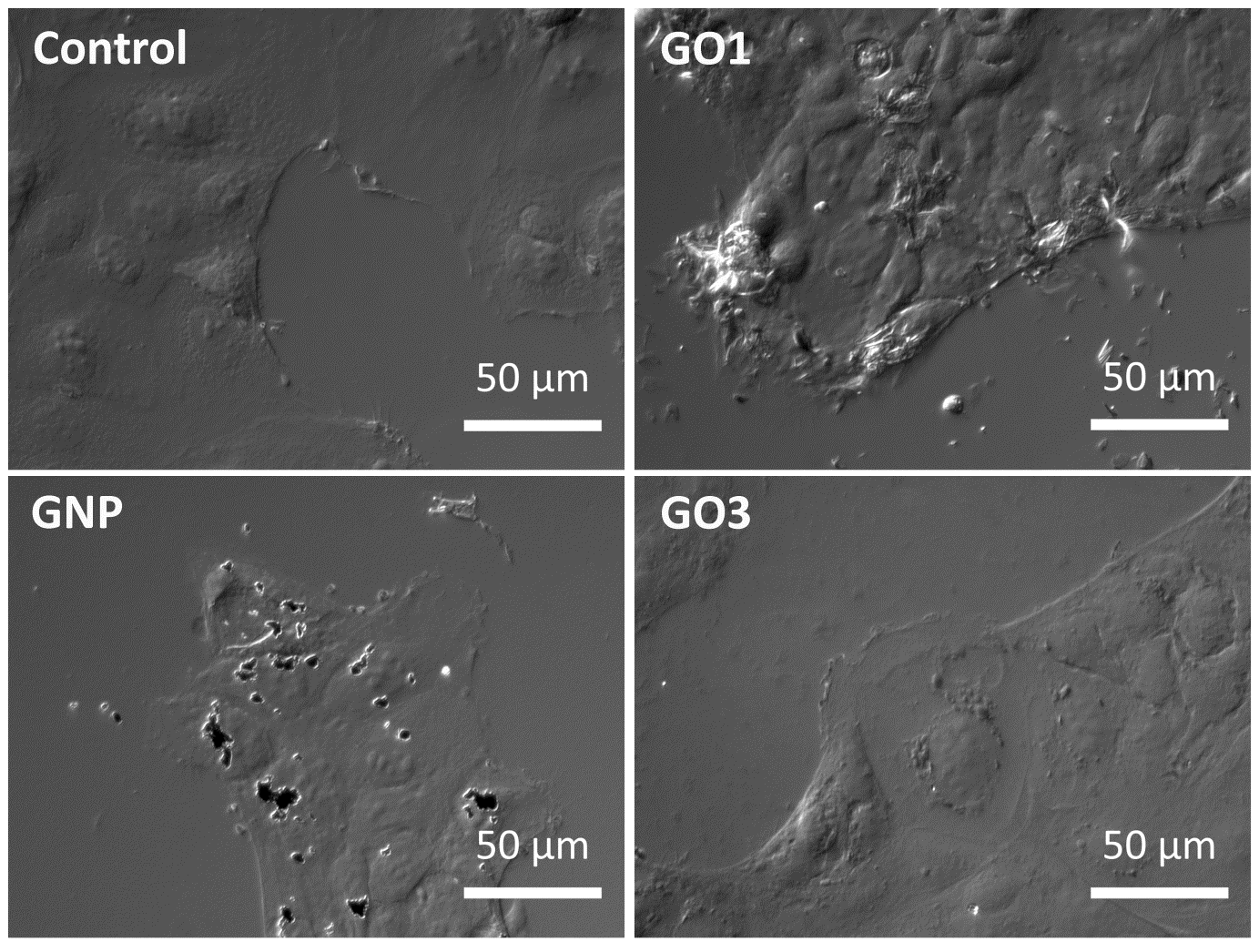


**Figure S2.** Fluorescence microscopy images (overlays) of non-confluent Caco-2 cells. Cells were exposed for 24 hours to 20 µg/ml GO1, GO3 or GNP respectively. Control cells not exposed to GO were run in parallel. Cell nuclei were labelled with DAPI (blue; λex = 335-383 nm, λem = 420-470 nm). Actin-network was labelled with Phalloidin-Alexa Fluor® 488 (green; λex = 455-495 nm, λem = 505- 555 nm). GRM is visible by transmitted differential interference contrast (TL DIC) microscopy. Microscopy analysis was performed with an Axio ImagerZ.1 microscope (Zeiss, Oberkochen, Germany).


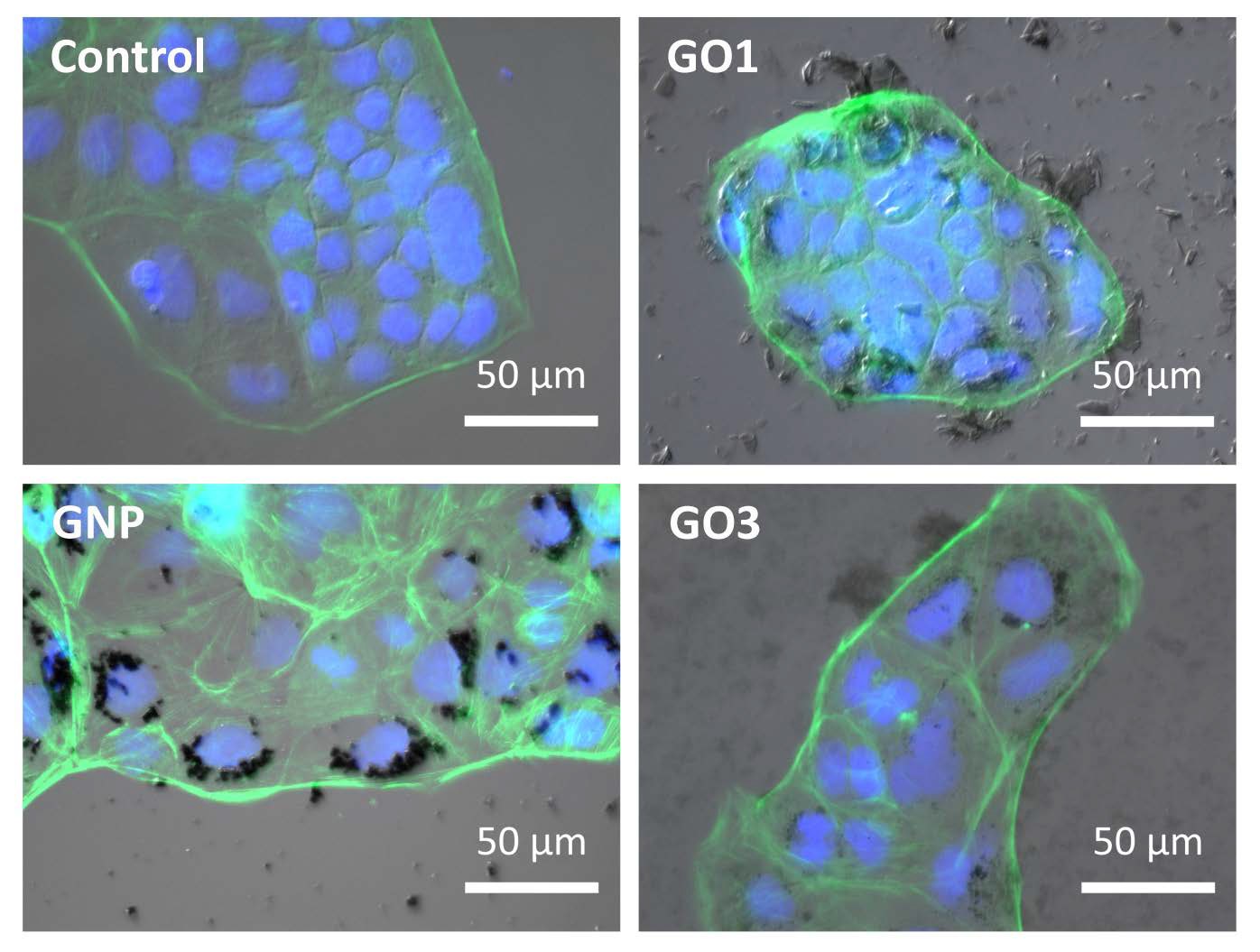


**Figure S3.** SEM images of non-confluent Caco-2 cells after exposure to GO1 or GO3 for 24 hours.

Cells were exposed to 40 µg GO1/ml (top, left) or 20 µg GO1/ml (top, right). GO1 sheets exhibited either highly crumpled morphology especially at the cell-substrate border or were aligned parallel to the cell surface. GO3 was applied in a concentration of 40 µg GO3/ml. GO3 is visible in form of mat- like agglomerates of folded and wrinkled sheets both on the substrate and cell surface (bottom, right). Formation of circular wave-like protrusions on the surface of GO3-exposed cells give hints towards the possible uptake mechanism macropinocytosis (bottom, left).


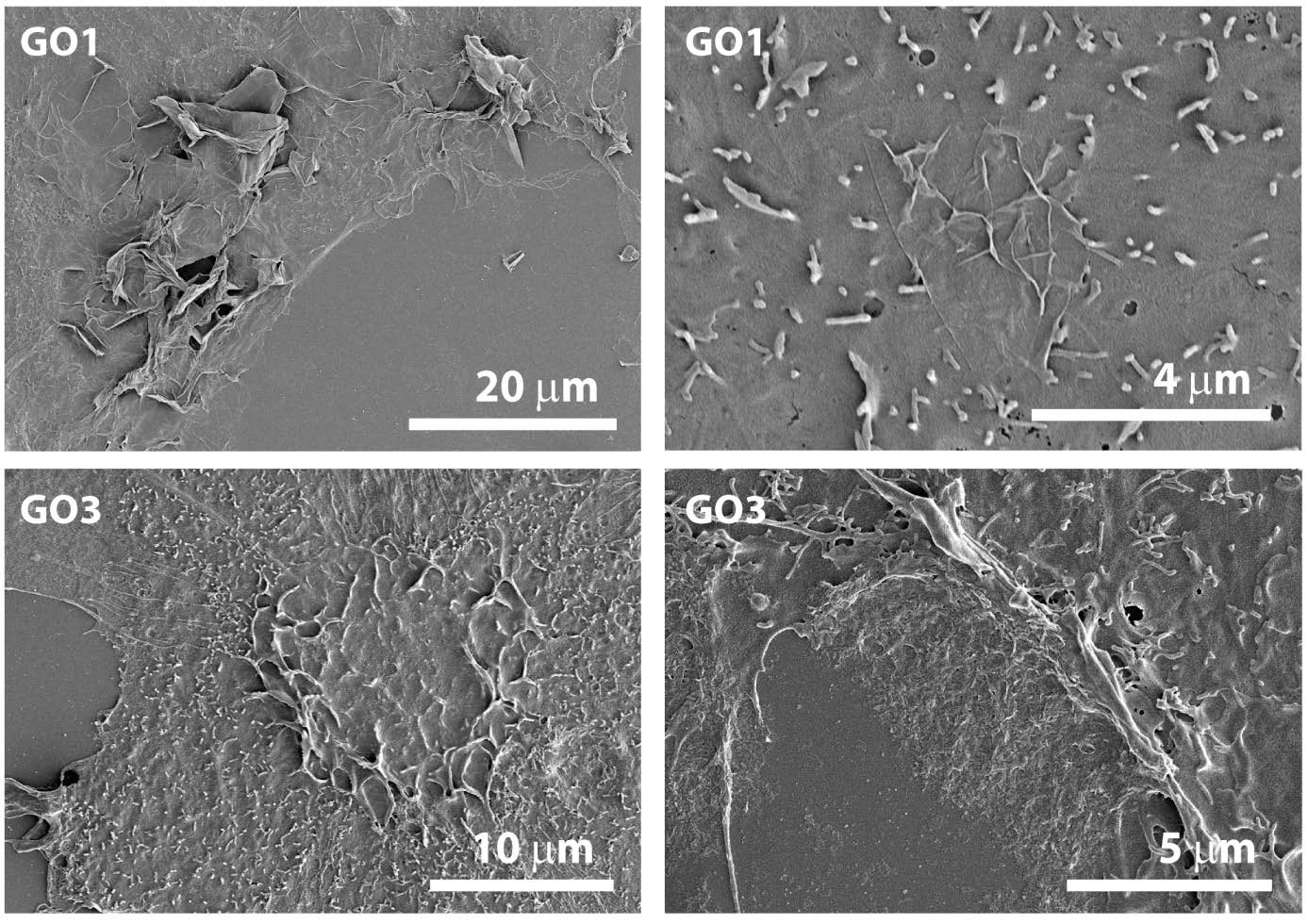


**Figure S4.** Interaction of GNP and the surface of non-confluent Caco-2 cells. SEM images of cells

after exposure to 20 µg GNP/ml for 24 h. Most of the shown GNP aggregates were found on the cell surface near the edges of Caco-2 islets and were associated with membrane protrusions. Bottom images: GNP aggregate is exemplarily displayed in purple to facilitate identification.


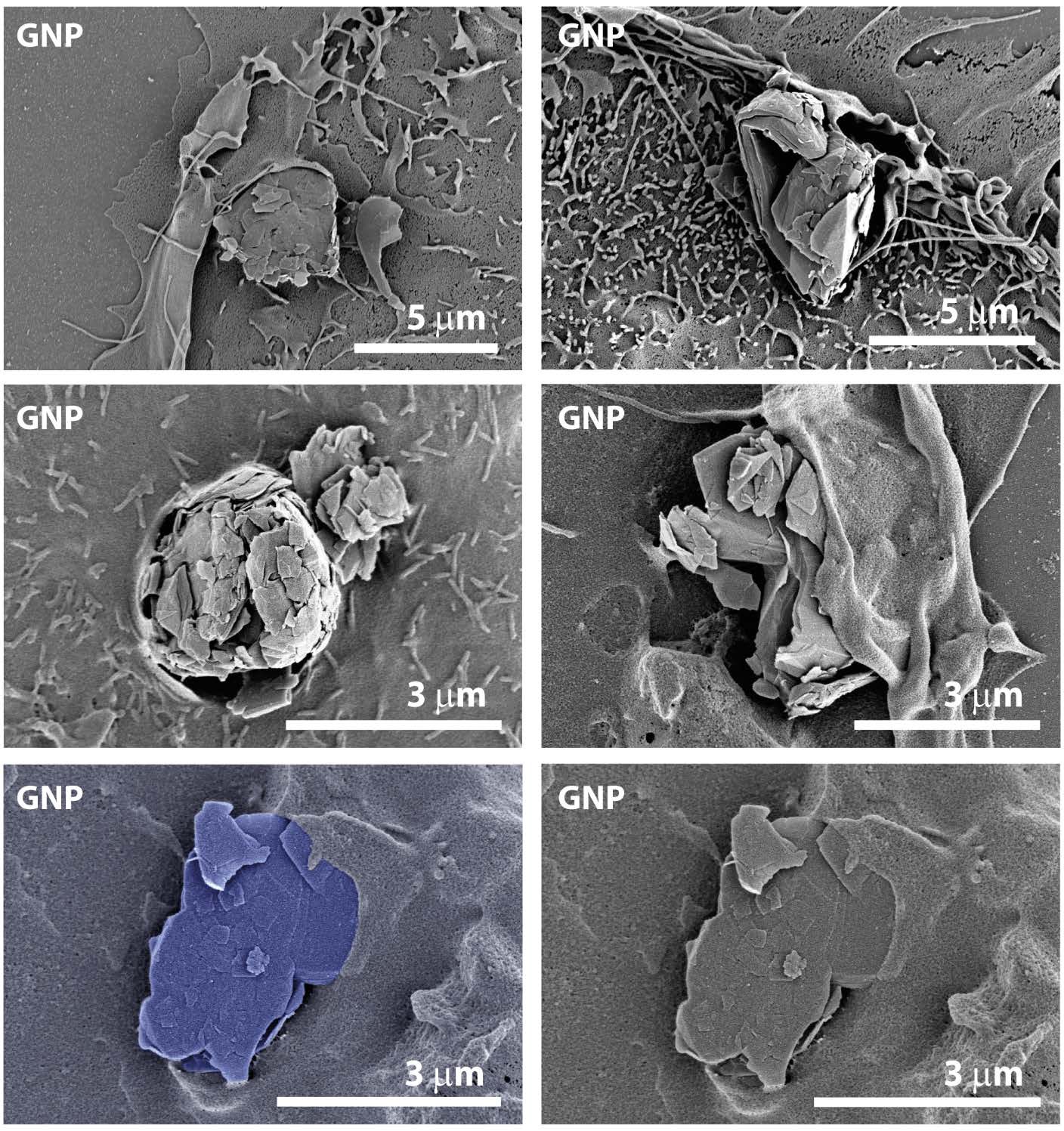


**Figure S5.** FACS analysis. Scatter plots of non-confluent Caco-2 cells after 24 h exposure to GRM.


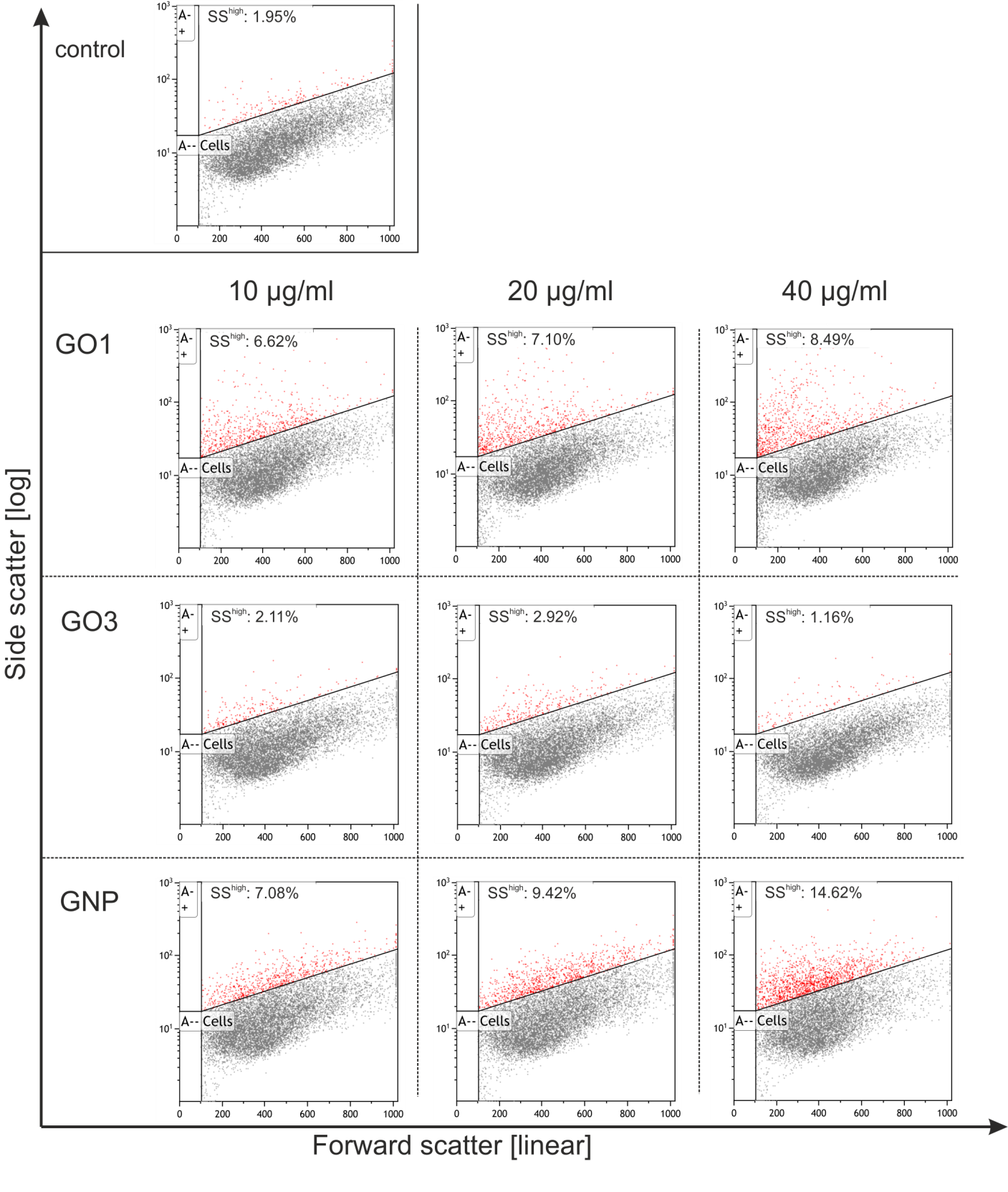


**Figure S6**. Cell surface morphology of differentiated Caco-2 cells. SEM images of control cells

without GO exposure and cells after exposure to 20 µg GO1/ml for 24 h. Only a few GO1 sheets could be identified on top of the brush border (highlighted by red boxes).


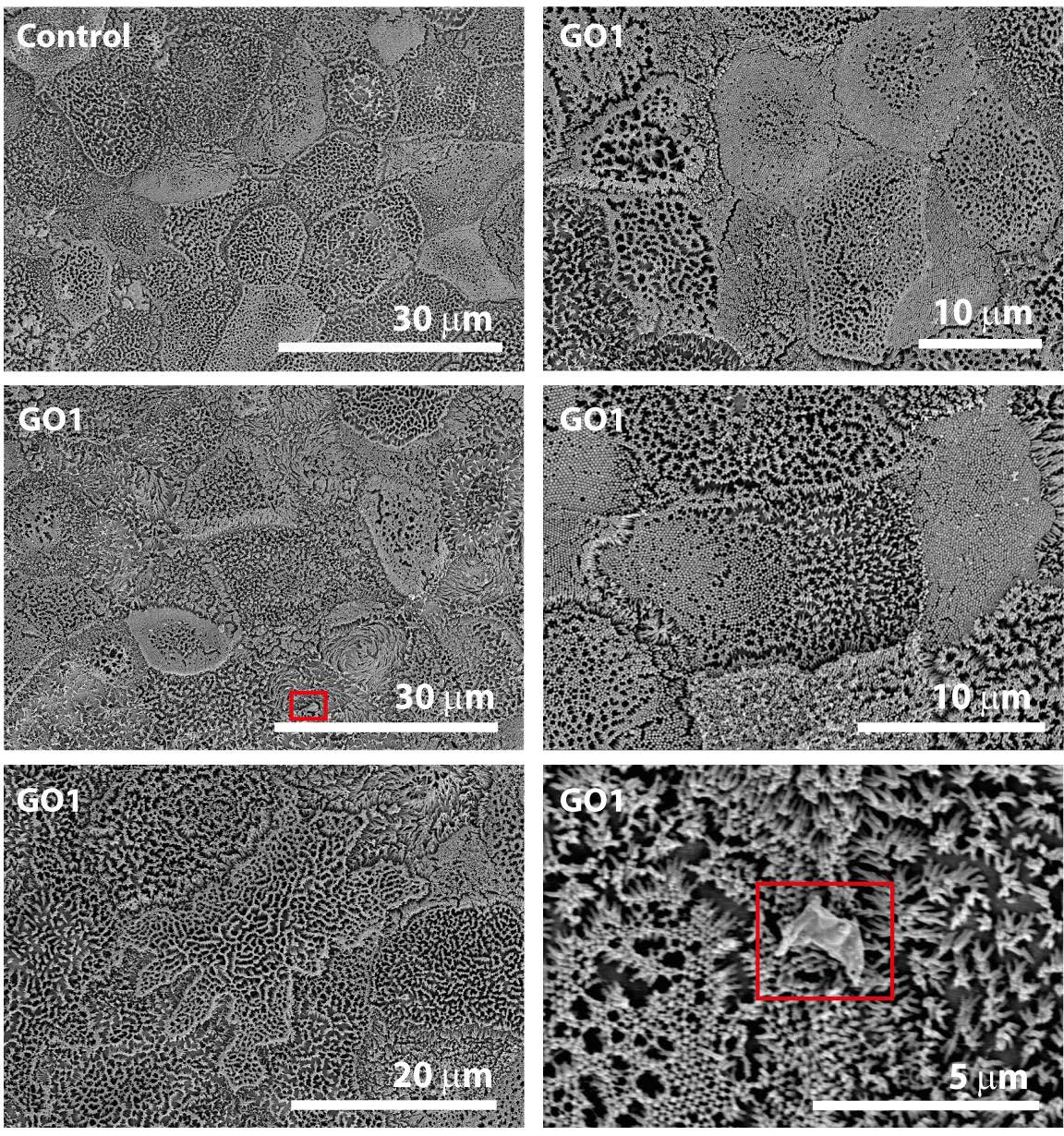


**(Immuno)fluorescence labeling of differentiated Caco-2 cells**

Caco-2 cells grown and differentiated on permeable supports (ThinCert™, Greiner bio-one, 12-well, polyethylene terephthalate (PET) membrane with 3.0 µm pore size) were exposed to the respective GO (GO1 or GO3) in supplemented cell culture medium for 24 hours. After exposure Caco-2 cells were washed twice in pre-warmed phosphate buffered saline (PBS), fixed in 4% paraformaldehyde in PBS and permeabilized with 0.9% Triton X-100 in PBS. Samples were washed twice in PBS and incubated in 5% BSA in PBS for 30 minutes. Tight junctions were labeled with ZO-1 mouse monoclonal antibody-Alexa Fluor® 488 (ZO1-1A12, Isotype Mouse IgG1 k, Catalog no. 339188, Invitrogen Corporation, 542 Flynn Rd, Camarilla, CA 93012, USA) in 1% BSA in PBS for 1 hour at room temperature and protected from light. After labeling samples were washed twice in PBS and further labeled with DAPI (4′,6-Diamidin-2-phenylindol, Catalog no. 9542, Sigma) and Phalloidin- Alexa Fluor® 546 (Catalog no A22283, Invitrogen, Molecular Probes, Eugene, Oregon, USA) for 20 minutes. Samples were washed three times in PBS. Transwell membranes with cells were carefully cut-out the housing and transferred to glass microscopy slides. Samples were mounted in Mowiol® 4- 88, covered by glass cover slides and dried over night at room temperature and protected from light. Microscopy analysis was performed by fluorescence microscope (Axio ImagerZ.1, Zeiss, Oberkochen, Germany) with Plan-Neofluar 20x/0.50 Objective. Representative images are shown in Figure S6.

**Figure S7.** Fluorescence microscopy images of differentiated Caco-2 cell monolayer. Control cells

without GO exposure and Caco-2 cells after exposure to GO; cells were exposed for 24 hours to 20 µg GO1/ml or 20 µg GO3/ml respectively. Channel 1 (Ch1; λex = 335-383 nm, λem = 420-470 nm) shows cell nuclei labelled with DAPI (blue). Channel 2 (Ch2; λex = 455-495 nm, λem = 505-555 nm) shows the presence of tight junctions by labelling with ZO-1 mouse monoclonal antibody-Alexa Fluor® 488 (green). Channel 3 (Ch3; λex = 538-562 nm, λem = 570-640 nm) shows the actin-network labelled with Phalloidin-Alexa Fluor® 546.


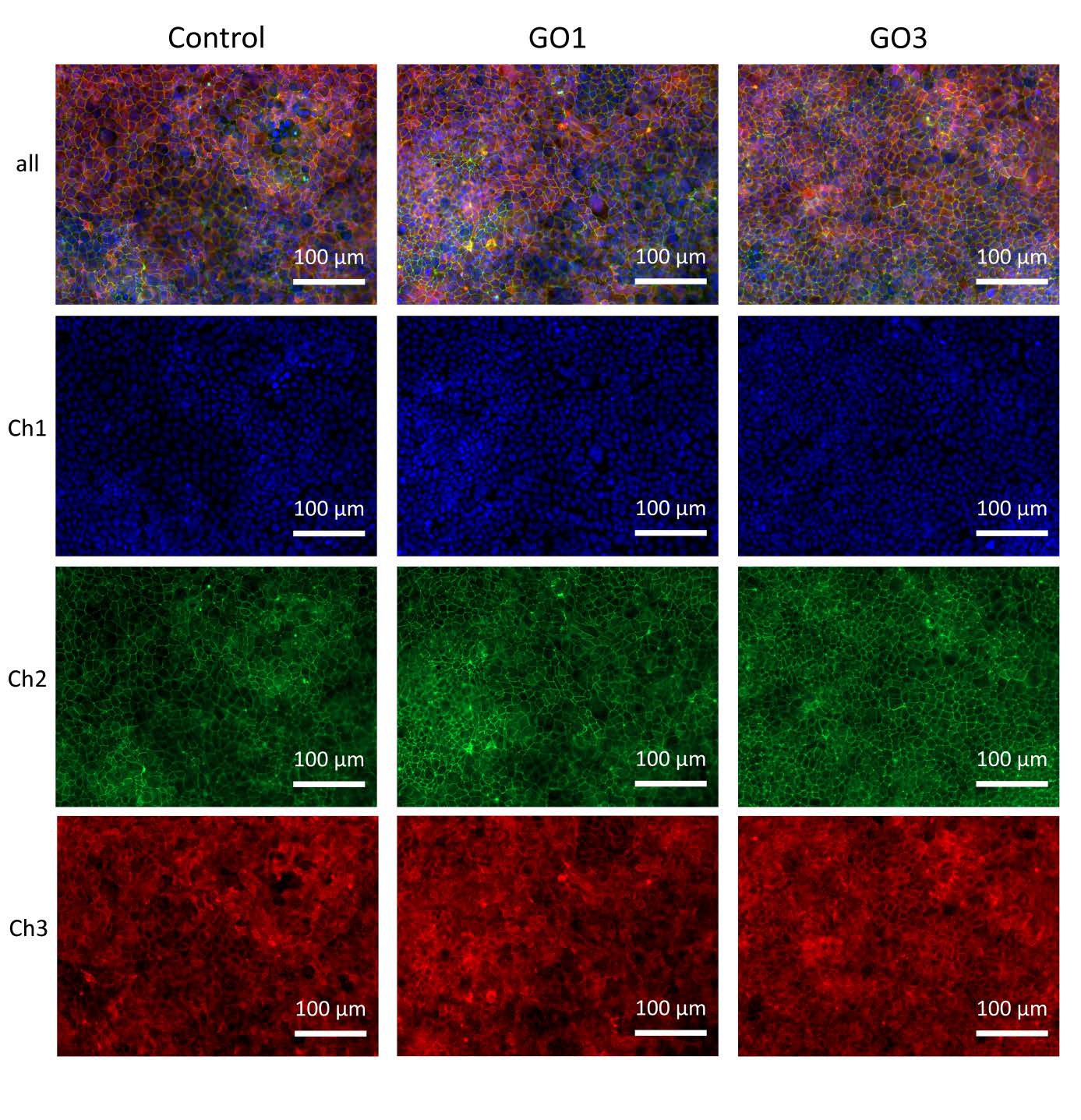

Supplement: Supplementary file 1 — Additional file 1: Figure S1. Differential interference contrast (DIC) images of non-confluent Caco-2 cells grown on glass cover slips. Cells were exposed to either 20 μg/ml GO1, GO3 or GNP for 24 hours. GRM-exposed cells showed no morphological differences in comparison to the unexposed control cells. Accumulation of dark material in the perinuclear region gives hints towards uptake of GO3 by non-confluent Caco-2 cells. Microscopy analysis was performed with an Axio ImagerZ.1 microscope (Zeiss, Oberkochen, Germany). Figure S2. Fluorescence microscopy images (overlays) of non-confluent Caco-2 cells. Cells were exposed for 24 hours to 20 μg/ml GO1, GO3 or GNP respectively. Control cells not exposed to GO were run in parallel. Cell nuclei were labelled with DAPI (blue; λex = 335-383 nm, λem = 420-470 nm). Actin-network was labelled with Phalloidin-Alexa Fluor® 488 (green; λex = 455-495 nm, λem = 505-555 nm). GRM is visible by transmitted differential interference contrast (TL DIC) microscopy. Microscopy analysis was performed with an Axio ImagerZ.1 microscope (Zeiss, Oberkochen, Germany). Figure S3. SEM images of non-confluent Caco-2 cells after exposure to GO1 or GO3 for 24 hours. Cells were exposed to 40 μg GO1/ml (top, left) or 20 μg GO1/ml (top, right). GO1 sheets exhibited either highly crumpled morphology especially at the cell-substrate border or were aligned parallel to the cell surface. GO3 was applied in a concentration of 40 μg GO3/ml. GO3 is visible in form of mat-like agglomerates of folded and wrinkled sheets both on the substrate and cell surface (bottom, right). Formation of circular wave-like protrusions on the surface of GO3-exposed cells give hints towards the possible uptake mechanism macropinocytosis (bottom, left). Figure S4. Interaction of GNP and the surface of non-confluent Caco-2 cells. SEM images of cells after exposure to 20 μg GNP/ml for 24 h. Most of the shown GNP aggregates were found on the cell surface near the edges of Caco-2 islets a [file 12951_2017_280_MOESM1_ESM.docx]
